# Revealing the Satellite DNA History in *Psalidodon* and *Astyanax* Characid Fish by Comparative Satellitomics

**DOI:** 10.3389/fgene.2022.884072

**Published:** 2022-06-21

**Authors:** Caio Augusto Gomes Goes, Rodrigo Zeni dos Santos, Weidy Rozendo Clemente Aguiar, Dálete Cássia Vieira Alves, Duílio Mazzoni Zerbinato de Andrade Silva, Fausto Foresti, Claudio Oliveira, Ricardo Utsunomia, Fabio Porto-Foresti

**Affiliations:** ^1^ Laboratório de Genética de Peixes, Faculdade Estadual Paulista “Júlio de Mesquita Filho”, Departamento de Ciências Biológicas, Faculdade de Ciências, Bauru, Brazil; ^2^ Instituto de Ciências Biológicas e da Saude, Universidade Federal Rural do Rio de Janeiro, Seropédica, Brazil; ^3^ Laboratório de Biologia e Genética de Peixes, Faculdade Estadual Paulista “Júlio de Mesquita Filho”, Instituto de Biociências, Botucatu, Brazil

**Keywords:** satellitome, satDNA evolution, characiforms, fish, neotropical fishes

## Abstract

Eukaryotic genomes are usually enriched in repetitive DNA sequences, which can be classified as dispersed or tandemly repeated elements. Satellite DNAs are noncoding monomeric sequences organized in a head-to-tail fashion that are generally located on the subtelomeric and/or pericentromeric heterochromatin. In general, a single species incorporates a diverse group of satellite DNA families, which collection is called satellitome. Here, we characterized three new satellitomes from distinct characid fish (*Psalidodon fasciatus, P. bockmanni,* and *Astyanax lacustris*) using a combination of genomic, cytogenetic, and bioinformatic protocols. We also compared our data with the available satellitome of *P. paranae.* We described 57 satellite DNA (satDNA) families of *P. fasciatus* (80 variants), 50 of *P. bockmanni* (77 variants), and 33 of *A. lacustris* (54 variants). Our analyses demonstrated that several sequences were shared among the analyzed species, while some were restricted to two or three species. In total, we isolated 104 distinctive satDNA families present in the four species, of which 10 were shared among all four. Chromosome mapping revealed that the clustered satDNA was mainly located in the subtelomeric and pericentromeric areas. Although all *Psalidodon* species demonstrated the same pattern of clusterization of satDNA, the number of clusters per genome was variable, indicating a high dynamism of these sequences. In addition, our results expand the knowledge of the As51 satellite DNA family, revealing that *P. bockmanni* and *P. paranae* exhibited an abundant variant of 39 bp, while *P. fasciatus* showed a variant of 43 bp. The majority of satDNAs in the satellitomes analyzed here presented a common library repetitive sequence in *Psalidodon* and *Astyanax*, with abundance variations in each species, as expected for closely related groups. In addition, we concluded that the most abundant satDNA in *Psalidodon* (As51) passed through a diversification process in this group, resulting in new variants exclusive of *Psalidodon*.

## Introduction

A significant part of eukaryotic genomes is composed of repetitive DNA sequences (Charlesworth et al., 1994), represented by transposable elements and tandemly arrayed sequences, such as multigene families and satellite DNAs (satDNAs) (Charlesworth et al., 1994; Jurka et al., 2005). SatDNAs are noncoding sequences organized in tandem arrays of up to hundreds of thousands of nucleotides (López-Flores and Garrido-Ramos, 2012; Plohl et al., 2012) that are typically observed in subtelomeric and pericentromeric heterochromatin areas, although short arrays of satDNAs dispersed in euchromatin have been documented (Kuhn, 2015; Ruiz-Ruano et al., 2016; Garrido-Ramos, 2017; Pita et al., 2017; Rodrigues et al., 2019; Montiel et al., 2021). They are originated from duplication of a new sequence by replication slippage or rolling circle replication, with posterior dispersion throughout the genome and massive local amplification (Ruiz-Ruano et al., 2016; Vondrak et al., 2020).

The evolution of satDNAs is characterized by a process called concerted evolution (Elder and Turner, 1995; Lorite et al., 2017). In this sense, satDNAs go through a step of homogenization, following mechanisms such as unequal crossing over and gene conversion (Smith, 1976; Dover, 1986), with homogenized variants fixed in populations by reproduction. In addition, related species may share an ancestral set of different conserved satDNA families (Fry and Salser, 1977; Ruiz-Ruano et al., 2016), according to the library hypothesis. Different variants may be amplified or depleted in each species, generating different collections of detectable satDNAs. Therefore, a part of the library may appear as an abundant satDNA, while others remain at low amounts (Camacho et al., 2022). To name this whole collection of satDNA families observed in a single genome, Ruiz-Ruano et al. (2016) proposed the term satellitome.

The first satellitome of a Neotropical fish was described for *Psalidodon paranae*, with 45 satDNA families (Silva et al., 2017), and some were detected by Fluorescence *in situ* hybridization (FISH) in congeneric species, corroboration of the library hypothesis. This identification of common satDNAs in three species of *Psalidodon* (Silva et al., 2017) and the recent diversification and phylogenetic proximity between *P. paranae*, *P. fasciatus*, and *P. bockmanni*, incites interest in this group for the study of the evolution of satDNAs. *Psalidodon* was part of the *Astyanax*, until recently (Terán et al., 2020). Prior to the description of the *P. paranae* satellitome, the only known satDNA in this group was As51 (Mestriner et al., 2000), which is widely used as a cytogenetic marker, and present in several species of *Psalidodon*, usually in subtelomeric chromatin regions (Abel et al., 2006; Kantek et al., 2009). In the *P. paranae* satellitome, As51 represents the most abundant satDNA, corresponding to ApaSat01-51 (Silva et al., 2017).

The satellitome of *P. paranae* is unique to the genus *Psalidodon*, although some of its satDNAs are observed in other related species (Silva et al., 2017). Previous evolutionary studies involving the characterization of the entire satellitome of a species are scarce. The objective of this study was to conduct, for the first time, a comparison among the catalogs of satDNAs, involving the satellitome of *P. paranae*, and three new satellitomes: *P. fasciatus*, *P. bockmanni*, and *A. lacustris*, to expand the knowledge of *Psalidodon* satDNA families and to describe the *Astyanax* satellitome for the first time.

## Materials and Methods

### Sampling and Cytogenetic Analyses

We analyzed individuals of *P. bockmanni*, *P. fasciatus*, and *A. lacustris* that were collected from natural populations of the Alambari (22°27′07.4"S; 4914′30.4″W) (*P. bockmanni* and *P. fasciatus*), Araras (22°27′49^″^S; 47°44′44^″^W) (*P. fasciatus*), and Batalha (22°23′40.8"S; 4906′34.7″W) (*A. lacustris*) rivers. Five individuals of each species were collected, with exception of *P. fasciatus*, in which we collected five individuals of each population analyzed (Alambari and Araras). The samples were collected, maintained, and analyzed following the protocols for the care and use of animals of the Brazilian Society for Laboratory Animal Science (SBCAL), and approved (protocol 1227) by Bioscience Institute/UNESP Ethics Committee on the Use of Animals (CEEAA/IBB/UNESP). The samples were collected with authorization from the relevant organizations (MMA/IBAMA/ICMBio/SISBIO—18884-1, registered with IBAMA No. 2567470). The individuals were deposited at the fish collection of Laboratório de Genética de Peixes, at UNESP, Bauru, São Paulo, Brazil, under the vouchers LGP12524—LGP12529 (*P. bockmanni*), LGP13006 and LGP14052—LGP14055 (*A. lacustris*), LGP12554—LGP12558 (*P. fasciatus*–Alambari), and LGP85536 (*P. fasciatus–*Araras).

The cytogenetic analysis to obtain mitotic chromosomes followed a protocol previously established (Foresti et al., 1981), using cells of anterior kidney tissue.

### DNA Extraction and Whole Genome Sequencing

We extracted total genomic DNA from the livers of *P. bockmanni*, *P. fasciatus* (Alambari, 2n = 46)*,* and *A. lacustris* using the Wizard Genomic Kit (Promega, Madison, United States), following the manufacturer’s instructions. RNA was removed using RNase A (Invitrogen, Waltham, United States). Genomic DNA sequencing from an individual of each of *P. fasciatus*, *P. bockmanni*, and *A. lacustris* was performed using an Illumina MiSeq (paired-end 2 × 250 bp). In addition, the library of *P. paranae* was used in our analyses (Silva et al., 2017).

### Satellitome Characterization and Additional Analyses

After quality and adapter trimming using Trimmomatic v0.33 (Bolger et al., 2014), we performed a high-throughput analysis of satDNAs within the genomes of *P. fasciatus*, *P. bockmanni*, and *A. lacustris*, using the satMiner bioinformatic protocol (Ruiz-Ruano et al., 2016). We performed a clustering of 2 × 500,000 reads, randomly selected using RepeatExplorer (Novák et al., 2013) with the default options to select clusters with a structure typical of satDNA. A search for contigs with tandem repetitions was performed using the dotplot tool, integrated into Geneious 8.1 software (Biomatters). We used the Deconseq software (Schmieder and Edwards, 2011) to filter the assembled contigs of all clusters designated as *in tandem* by RepeatExplorer, and the remaining sequences were clustered using RepeatExplorer. These processes were repeated until no new satDNA sequences appeared.

Subseqently, we filtered the obtained sequences and removed other tandemly repeated elements, such as multigene families, and used the software rm_homology (https://github.com/fjruizruano/satminer/blob/master/rm_homology.py) to eliminate redundancies of isolated contigs, and to group the sequences into the same variant, different variants of the same family, or superfamilies (similarities greater than 95, 80, and 50%, respectively), as established by Ruiz-Ruano et al., 2016. The same method was used to compare the satellitomes of *P. paranae*, *P. fasciatus*, *P. bockmanni*, and *A. lacustris*. The abundance and divergence of each satDNA variant were estimated using RepeatMasker software (Smit et al., 2017), using randomly selected reads (2 × 5,000,000 reads). In RepeatMasker, the reads of species were separately mapped against concatenated monomers of satDNAs consensus sequences (spanning 200 nucleotides). SatDNA families were named according to a previous study (Ruiz-Ruano et al., 2016), with the species abbreviations Pfa, Pbo, and Ala, for *P. fasciatus*, *P. bockmanni*, and *A. lacustris*, respectively, in addition to the term “Sat” and a catalog number in order of decreasing abundance. The catalogs of satDNA families were deposited on the GenBank with accession numbers OM793143-OM793191 (*P. bockmanni*), OM793192-OM793247 (*P. fasciatus*), and OM793248-OM793279 (*A. lacustris*). In addition, we generated repeat landscapes to estimate the average divergence, considering the distances between sequences based on the Kimura-2-parameter model using the script calcDivergenceFromAlign.pl, of the RepeatMasker suite (Supplementary Figures S1–S3).

To better understand the satDNA families observed in all four satellitomes described, we generated variant profiles and coverage depths using the RepeatProfiler pipeline (Negm et al., 2021) to analyze the sequence variation in the genomes studied. In addition, we included a library of *A. mexicanus*, a species model of the *Astyanax* genus, obtained in NCBI, SRA database under the access number SRR6386652. We randomly selected 2 × 1,000,000 reads for all five species, and the target satDNA families were concatenated to a minimum of 200 bp. We used dimers when the satDNA were greater than 200 bp. Read mapping was performed with Bowtie2 (Langmead and Salzberg, 2012) with the preset values “-sensitive” and “no-mixed.” We utilized 10 single-copy fish genes to be mapped for single-copy normalization of the read coverage, as described previously (dos Santos et al., 2021). The genes used here were *ppfia1* (XM_022685633.1), *foxl2* (XM_007232295.3), *prospero* (XM_017708821.1), *msh4* (XM_017711771.1), *zdhhc22* (XM_017711775.1), *coq6* (XM_017711829.1), *znf106* (XM_017711848.1), *lactamase* (XM_022682177.1), *gastrula zinc finger* (XM_022685636.1), and *tubulin kinase* (XM_017711762.1).

After verifying a satDNA family related to the cytogenetic marker As51 on the satellitome of *P. bockmanni* (PboSat03-39), characterized by a deletion of 12 bp, we investigated the presence of this variant in the genomes of *P. paranae*, *P. bockmanni*, *P. fasciatus*, *A. lacustris*, and *A. mexicanus*. In addition, a variant of 43 bp was observed in the satellitome of *P. fasciatus*, and we included this sequence in our analysis. We collected monomers of the three variants cited (51 bp, 39 bp, and 43 bp) from the genomes of the five species, using a random selection of 2 × 250,000 reads for each species. We then aligned the isolated reads against each satDNA variant, to only select full reads (Utsunomia et al., 2019). Subsequently, we discarded singletons using CD-HIT software (Li and Godzik, 2006). Due to the large number of monomers obtained, principally in *P. paranae*, we performed a random proportional selection of monomers for each species using Seqtk software (https://github.com/lh3/seqtk). A total of 2044 monomers were utilized, and information on the monomers obtained for each species and the quantity of monomers utilized in our analyses are shown in Supplementary Table S1. Finally, we constructed a minimum spanning tree (MST) of the pairwise differences and considered the relative abundance of the haplotypes using software PHYLOViZ 2.0 (Nascimento et al., 2017). The images were produced using the Inkscape software.

### Fluorescence *in situ* Hybridization

FISH experiments were performed with eight satDNA families, which were common to the four analyzed species. We utilized primers described by Silva et al. (2017) and probes were labeled with digoxigenin-11dUTP in PCR reactions. FISH experiments were performed following the protocol established by Pinkel et al. (1986), with some modifications (Utsunomia et al., 2017). The metaphasic plate was treated with RNase A (50 μg/ml), for 50 min, with subsequent chromosomal DNA denaturation in 70% formamide/2 × SSC for 2 min, at 70°C. After hybridization, the slides were washed in 0.2 × SSC/15% formamide for 5 min at 42°C, with subsequent washes in 4 × SSC/0.5% Tween-20, at room temperature. Probe detection was performed with anti-digoxigenin-rhodamine (Roche, Basiléia, Switzerland) and the chromosomes were counterstained with DAPI (4ʹ,6-diamino-2-phenylindole, Vector Laboratories, Burlingame, United States). The results were analyzed using an optical microscope (Olympus BX61). Images were captured using the DP Controller software (Olympus®, Hamburg, Germany).

## Results

### Cytogenetic Analysis

Five individuals from each species were collected. Diploid numbers observed were consistent with the literature, with 2n = 50 for *P. bockmanni* and *A. lacustris*. Populations of *P. fasciatus* demonstrated differential diploid numbers, with 2n = 46 for those from the Alambari River and 2n = 48 for the Araras River. The karyotype formulas were 3m+5sm+6st+11a for *P. bockmanni*, 3m+5sm+12st+4a for *P. fasciatus* (Araras), 4m+7sm+9st+3a for *P. fasciatus* (Alambari), and 3m+9sm+9st+4a for *A. lacustris*. The species *P. fasciatus* is part of a “species complex”, with diploid numbers varying between 45 and 49, so our results are consistent with the literature (Kantek et al., 2009; Pasa et al., 2021). None of the analyzed individuals had B chromosomes.

### Description of Two New Satellitomes of *Psalidodon* and the First Satellitome of *Astyanax*

After several iterations with the satMiner toolkit protocol (6 for *P. fasciatus*, seven for *P. bockmanni*, and three for *A. lacustris*), until no satDNA was uncovered, we found 57 families of satDNAs for *P. fasciatus* (80 variants), 50 for *P. bockmanni* (77 variants) and 33 for *A. lacustris* (54 variants). The length distribution of satDNA families revealed the predominance of short satDNAs (<100 bp) for *P. fasciatus* (42) and *P. bockmanni* (35), corresponded to 73.9 and 70.0% the satellitomes, respectively. In contrast, long satDNAs predominated in *A. lacustris* (18), corresponding to 54.5% of the satellitome. The repeat unit length (RUL) ranged from 6 to 286 bp for *P. fasciatus* (median 82.08); 6 to 584 for *P. bockmanni* (median 111.3) and 6 to 3028 in *A. lacustris* (median 316.27). The A + T content varied between 33.9 and 78.2% for *P. fasciatus*, (median 62.1%); 32.1–72.4% in *P. bockmanni* (median 57.9%), and 40.0–71.2% in *A. lacustris* (median 60.4%), indicating a predominance of A + T-rich satDNAs. Complete information on the three new satellitomes is presented in Tables 1–3. The Shapiro-Wilks test demonstrated that only the A + T content showed a normal distribution (W = 0.973, *p* = 0.235 for *P. fasciatus*, W = 0.984, *p* = 0.34 for *P. bockmanni*, and W = 0.945, *p* = 0.115 for *A. lacustris*). Kendall’s rank correlation test demonstrated that the only correlation between traces was a negative correlation between RUL and divergence in *A. lacustris* (tau = −0.465).

**TABLE 1:** Main characteristics of *Psalidodon fasciatus* satellitome.

satDNA family	RUL	A + T (%)	V	Abundance	Divergence (%)
PfaSat01-51	51	58.8	4	0.09111751	6.16
PfaSat02-237	237	64.5	1	0.03233236	5.25
PfaSat03-97	97	54.6	3	0.02888047	13.62
PfaSat04-51	51	54.9	1	0.01037053	14.5
PfaSat05-71	71	54.9	2	0.01018242	18.04
PfaSat06-85	85	57.6	1	0.00988488	13.97
PfaSat07-31	31	64.9	2	0.00987813	19.69
PfaSat08-42	42	57.1	2	0.00919605	14.18
PfaSat09-177	177	67.2	1	0.00684888	12.49
PfaSat10-61	61	70.4	1	0.00675362	5.6
PfaSat11-21	21	76.1	2	0.00635465	14.7
PfaSat12-68	68	61.7	1	0.00468916	4.77
PfaSat13-91	91	50.5	3	0.00423541	6.39
PfaSat14-40	40	57.5	1	0.00411315	3.02
PfaSat15-187	187	67.3	1	0.00402267	9.25
PfaSat16-61	61	70.4	1	0.00396132	3.81
PfaSat17-59	59	66.1	1	0.00374524	6.13
PfaSat18-27	27	66.6	3	0.00358966	6.42
PfaSat19-22	22	54.5	1	0.00342889	13.93
PfaSat20-76	76	68.4	3	0.00342035	7.47
PfaSat21-109	109	60.5	1	0.00335123	4.62
PfaSat22-24	24	70.8	1	0.00329913	7.18
PfaSat23-236	236	64.4	1	0.00315699	5.99
PfaSat24-83	83	51.8	1	0.00304989	9.26
PfaSat25-46	46	78.2	1	0.00269382	4.78
PfaSat26-54	54	46.2	2	0.00259426	10.15
PfaSat27-197	197	63.4	1	0.00234954	4.03
PfaSat28-51	51	54.9	1	0.00225298	6.8
PfaSat29-190	190	62.1	2	0.00218743	8.99
PfaSat30-85	85	57.6	1	0.00218497	10.27
PfaSat31-42	42	50.0	2	0.00205879	8.6
PfaSat32-65	65	66.1	2	0.00180669	4.15
PfaSat33-286	286	67.8	1	0.0018058	6.02
PfaSat34-103	103	72.8	1	0.00172698	7.39
PfaSat35-142	142	73.9	1	0.00160352	10
PfaSat36-33	33	75.7	1	0.00148574	5.83
PfaSat37-166	166	70.4	1	0.00145701	7.53
PfaSat38-52	52	71.1	2	0.001455	14.65
PfaSat39-100	100	66.0	1	0.00143516	12.24
PfaSat40-143	143	76.2	1	0.00141934	6.73
PfaSat41-6	6	50.0	1	0.00141474	20.4
PfaSat42-51	51	64.7	1	0.00141112	11.93
PfaSat43-191	191	64.9	1	0.00133484	8.45
PfaSat44-141	141	64.5	1	0.0012583	4.02
PfaSat45-41	41	63.4	1	0.00122238	12.8
PfaSat46-54	54	50.0	1	0.00113283	6.39
PfaSat47-35	35	68.5	2	0.00109669	8.86
PfaSat48-27	27	74.0	2	0.00101737	10
PfaSat49-42	42	42.8	1	0.00096175	7.35
PfaSat50-29	29	51.7	1	0.00076152	6.71
PfaSat51-22	22	59.0	2	0.00073328	6.65
PfaSat52-21	21	57.1	1	0.00067703	5.45
PfaSat53-52	52	59.6	1	0.00067471	10.81
PfaSat54-56	56	33.9	1	0.00061054	4.88
PfaSat55-43	43	62.7	1	0.00057551	9.08
PfaSat56-55	55	67.2	1	0.00041117	4.47
PfaSat57-51	51	60.7	1	1.41E-05	20.46

**TABLE 2:** Main characteristics of *Psalidodon bockmanni* satellitome.

satDNA family	RUL	A + T (%)	V	Abundance	Divergence (%)
PboSat01-51	51	56.8	4	0.01690405	3.46
PboSat02-235	235	64.2	1	0.00606882	1.96
PboSat03-39	39	48.7	2	0.00547919	1.91
PboSat04-235	235	62.5	1	0.00132786	13.92
PboSat05-84	84	55.9	1	0.00115793	13.99
PboSat06-23	23	52.1	1	0.00083767	11.66
PboSat07-31	31	64.5	1	0.00079599	19.14
PboSat08-188	188	67.5	2	0.00075067	15.74
PboSat09-35	35	62.8	5	0.00071892	7.09
PboSat10-40	40	57.5	2	0.00064097	2.62
PboSat11-27	27	62.9	1	0.00057536	6.64
PboSat12-190	190	61.0	1	0.00053385	9.01
PboSat13-106	106	60.3	2	0.00048688	3.76
PboSat14-61	61	72.1	1	0.00046441	5.84
PboSat15-87	87	72.4	1	0.00043171	5.58
PboSat16-63	63	69.8	1	0.00039598	7.36
PboSat17-69	69	60.8	1	0.00038312	4.45
PboSat18-52	52	46.1	3	0.00035021	8.76
PboSat19-22	22	50.0	1	0.00032650	10.62
PboSat20-107	107	40.1	1	0.00032312	13.37
PboSat21-82	82	56.0	1	0.00030316	8.35
PboSat22-22	22	40.9	2	0.00029609	14.61
PboSat23-54	54	46.2	2	0.00028669	6.36
PboSat24-50	50	66.0	2	0.00027735	14.36
PboSat25-42	42	52.3	2	0.00026625	6.50
PboSat26-21	21	57.1	1	0.00025531	6.30
PboSat27-51	51	54.9	1	0.00024676	6.54
PboSat28-62	62	58.0	1	0.00023092	12.37
PboSat29-142	142	77.4	1	0.00022172	5.90
PboSat30-55	55	67.2	1	0.00020175	3.57
PboSat31-657	657	53.5	1	0.00019655	2.74
PboSat32-193	193	56.4	1	0.00016677	7.80
PboSat33-42	42	42.8	2	0.00015628	12.87
PboSat34-56	56	32.1	4	0.00015562	4.74
PboSat35-584	584	58.3	1	0.00015463	1.98
PboSat36-419	419	49.8	1	0.00014438	4.05
PboSat37-188	188	65.9	1	0.00014396	9.31
PboSat38-91	91	51.6	3	0.00014333	7.47
PboSat39-59	59	64.4	1	0.00014129	2.57
PboSat40-78	78	56.4	3	0.00014103	5.13
PboSat41-204	204	49.5	1	0.00013263	9.14
PboSat42-112	112	63.3	1	0.00012000	4.51
PboSat43-52	52	67.3	1	0.00011740	12.35
PboSat44-6	6	50.0	1	0.00011095	17.28
PboSat45-220	220	57.7	1	0.00010466	8.41
PboSat46-90	90	67.7	1	0.00010049	3.87
PboSat47-52	52	69.2	2	9.18E-06	6.15
PboSat48-32	32	50.0	1	8.58E-06	4.15
PboSat49-42	42	57.1	1	8.56E-06	7.17
PboSat50-62	62	66.1	2	7.25E-06	6.69

**TABLE 3:** Main characteristics of *Astyanax lacustris* satellitome.

satDNA family	RUL	A + T (%)	V	Abundance	Divergence (%)
AlaSat01-91	91	54.9	8	0.0019966	5.1
AlaSat02-186	186	64.5	1	0.00186961	0.98
AlaSat03-3028	3028	64.0	1	0.00191953	5.63
AlaSat04-151	151	63.5	2	0.00163407	2.49
AlaSat05-364	364	50.2	1	0.00164291	5.98
AlaSat06-42	42	54.7	1	0.00095201	14.46
AlaSat07-189	189	64.5	4	0.00095719	9.32
AlaSat08-236	236	63.9	1	0.00098154	12.74
AlaSat09-31	31	64.5	1	0.00093329	19.82
AlaSat10-84	84	57.1	2	0.0004786	15.78
AlaSat11-6	6	50.0	1	0.00046441	17.28
AlaSat12-177	177	66.6	1	0.00035606	16.02
AlaSat13-24	24	70.8	1	0.00031664	8.12
AlaSat14-62	62	70.9	2	0.00031979	7.96
AlaSat15-69	69	59.4	1	0.00027686	4.55
AlaSat16-251	251	56.9	1	0.00030137	11.4
AlaSat17-58	58	67.2	3	0.00028017	8.85
AlaSat18-80	80	71.2	1	0.00029079	9.31
AlaSat19-106	106	60.3	3	0.00022585	5.99
AlaSat20-85	85	57.6	2	0.0002287	13.25
AlaSat21-899	899	56.5	1	0.0002351	4.64
AlaSat22-22	22	45.4	2	0.00019073	14.02
AlaSat23-1242	1242	52.8	1	0.00022293	0.86
AlaSat24-577	577	58.7	1	0.00021396	2.13
AlaSat25-52	52	67.3	2	0.00017473	13.82
AlaSat26-418	418	52.3	1	0.00016463	4.59
AlaSat27-35	35	68.5	2	0.00013577	7.57
AlaSat28-574	574	64.9	1	0.00014314	5.37
AlaSat29-185	185	40.0	1	0.00013656	6.23
AlaSat30-352	352	69.3	1	0.00013661	8.13
AlaSat31-552	552	52.8	1	0.00013736	0.89
AlaSat32-187	187	68.4	3	0.00011427	9.54
AlaSat33-22	22	63.6	1	1.00E-07	29.07

Comparisons between satDNA families for each species detected homology among sequences. Four superfamilies were detected in *P. fasciatus*, three in *P. bockmanni,* and only one in *A. lacustris* (Table 4). In most cases, the variation between sequences involved in a superfamily was caused by nucleotide substitutions. However, deletions of segments from 8 to 10 bp were observed in one of the sequences of SF1 in *P. bockmanni* and SF1, SF3, and SF4 in *P. fasciatus* (Supplementary Material).

**TABLE 4:** Superfamilies characterized in satellitomes of *Psalidodon fasciatus*, *Psalidodon bockmanni,* and *Astyanax lacustris*.

	SatDNA	SatDNA	SatDNA	Similarity
*P. bockmanni*				
SF1	PboSat01-51	PboSat03-39	-	68.27%
SF2	PboSat02-235	PboSat04-235	-	78.90%
SF3	PboSat06-23	PboSat22-22	-	65.21%
*P. fasciatus*				
SF1	PfaSat01-51	PfaSat55-43	PfaSat57-51	78.43%/69.23%
SF2	PfaSat02-237	PfaSat23-236	-	79.83%
SF3	PfaSat17-59	PfaSat42-51	-	60.65%
SF4	PfaSat22-24	PfaSat36-33	-	72.72%
*A. lacustris*				
SF1	AlaSat22-22	AlaSat33-22	-	72.72%

### Comparative Analysis Demonstrated the Conservation of Several Satellite DNA Families Between *Psalidodon* and *Astyanax*

We performed a comparative analysis between the three satellitomes described in this study and that of *P. paranae*, using the RepeatMasker software. Of a total of 104 satDNA families present in the four species, 10 were observed in all species analyzed. One of them were identified as the telomeric sequence (ApaSat07-06-tel) that was included in other fish satellitomes (Silva et al., 2017; Utsunomia et al., 2019; Stornioli et al., 2021), and other was identified as CharSat01-52 (ApaSat29-52), which was conserved of the several species in Characidae family (dos Santos et al., 2021). Eight other sequences were observed in all four species, with at least 50% similarity (Table 5). In addition, several sequences were detected in only two or three species analyzed, as shown in Table 6, along with their degrees of similarity.

**TABLE 5:** SatDNA families with at least 50% of similarity in three species of *Psalidodon* and one species of *Astyanax*.

*P. paranae*	*P. bockmanni*	*P. fasciatus*	*A. lacustris*
ApaSat02-236	Pbosat02-235/Pbosat04-235	Pfasat02-237/Pfasat23-236	Alasat08-236
Apasat03-91	Pbosat38-91	Pfasat13-91	Alasat01-91
Apasat04-233	Pbosat02-235/Pbosat04-235	Pfasat02-237/Pfasat23-236	Alasat08-236
Apasat07-6-tel	Pbosat44-6	Pfasat41-6	Alasat11-6
Apasat08-35	Pbosat09-35	Pfasat47-35	Alasat27-35
Apasat11-22	Pbosat19-22	Pfasat19-22	Alasat22-22/Alasat33-22
Apasat12-69	Pbosat17-69	Pfasat12-68	Alasat15-69
Apasat29-52	Pbosat43-52	Pfasat38-52	Alasat25-52
Apasat30-50	Pbosat24-50	Pfasat17-59/Pfasat42-51	Alasat17-58
Apasat40-189	Pbosat37-188	Pfasat43-191	Alasat07-189

**TABLE 6:** SatDNAs similarity in two or three species analyzed. Similarity in superfamilies level (between 50 and 80%) are highlighted (*).

*P. paranae*	*P. bockmanni*	*P. fasciatus*	*A. lacustris*
Apasat01-51	Pbosat01-51	Pfasat01-51	-
	Pbosat03-39*	Pfasat55-43*	
		Pfasat57-51*	
Apasat05-23	Pbosat06-23	-	-
Apasat13-23	Pbosat22-22*		
Apasat06-86	-	Pfasat24-83	-
Apasat09-21	-	Pfasat11-21	-
Apasat10-179	-	-	Alasat02-186
Apasat15-51	Pbosat27-51	Pfasat28-51	-
Apasat16-54	Pbosat18-52	Pfasat26-54	-
Apasat17-365	-	-	Alasat05-364
Apasat18-58	Pbosat23-54	Pfasat46-54	-
Apasat19-77*	Pbosat16-63*	-	-
Apasat22-62	Pbosat28-62	-	-
Apasat24-78	Pbosat40-78	-	-
Apasat27-178*	-	-	Alasat29-185*
Apasat33-112	Pbosat42-112	-	-
Apasat36-21	Pbosat26-21	Pfasat52-21	-
Apasat38-107	Pbosat20-107	-	-
Apasat39-32	Pbosat48-32	-	-
Apasat42-90	Pbosat46-90	Pfasat32-65*	-
-	Pbosat05-84	Pfasat06-85	Alasat10-84
-	Pbosat07-31	Pfasat07-31	Alasat09-31
-	Pbosat08-188*	Pfasat15-187	Alasat32-187
-	Pbosat10-40	Pfasat14-40	-
-	Pbosat11-27	Pfasat18-27	-
-	Pbosat12-190	Pfasat29-190	-
	Pbosat13-106	Pfasat21-109	Alasat19-106
-	Pbosat14-61	Pfasat10-61	Alasat14-62
-	Pbosat15-87	Pfasat20-76	Alasat18-80
-	Pbosat21-82	Pfasat30-85	Alasat20-85
-	Pbosat25-42	Pfasat31-42	-
-	Pbosat29-142	Pfasat40-143	-
-	Pbosat30-55	Pfasat56-55	-
-	Pbosat31-657	-	Alasat31-552
-	Pbosat33-42	Pfasat49-42	-
-	Pbosat34-56	Pfasat54-56	-
-	Pbosat35-584	-	Alasat24-577
-	Pbosat36-419	-	Alasat26-418
-	-	Pfasat08-42	Alasat06-42
-	-	Pfasat09-177	Alasat12-177
-	-	Pfasat22-24	Alasat13-24
		Pfasat36-33*	
-	-	Pfasat27-197*	Alasat04-151*

### RepeatProfiler Reveals Highly Conserved Satellite DNA Families Between Genera

**FIGURE 1:**
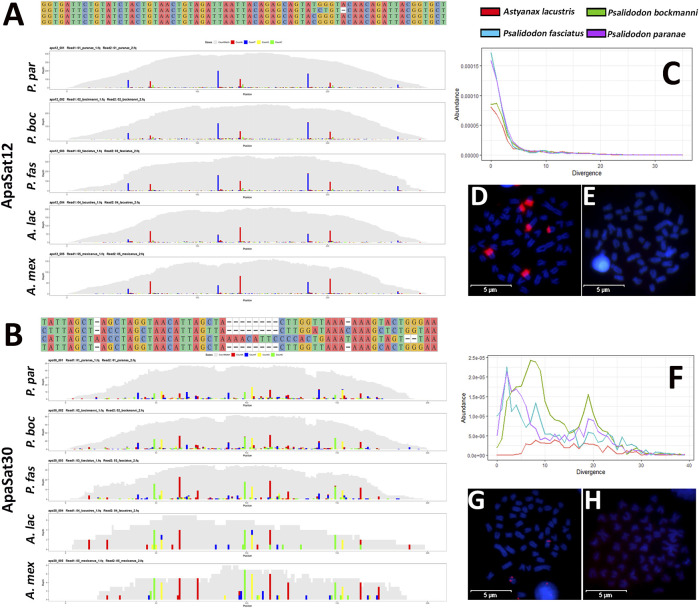
Repeats landscapes of conserved satDNA families conserved between Psalidodon and Astyanax. Alignments and repeat profilers of ApaSat12-69 **(A)** and Apasat30-50 **(B)**. Repeat landscapes of Apasat12-69 **(C)**, Apasat30-50 **(F)** demonstrate the abundance and divergence of these satDNAs in the analyzed species. In addition, these satDNAs demonstrated FISH signals in all Psalidodon species **(D and G)**, but not in Astyanax **(E and H)**.

We generated RepeatProfiler plots of the ten satDNAs shared between the four species. In addition, we included the genome of *Astyanax mexicanus* for this analysis. We represent profiles of ApaSat12-69, and ApaSat30-50 in Figure 1, and remaining are in Supplementary Material. Our results revealed a high degree of conservation of ApaSat11-22 and ApaSat12-69 for all five species analyzed, with a similar degree of abundance in all groups. As expected, in all cases, the profiles demonstrated greater similarity between species of the *Psalidodon* than those of the *Astyanax* (ApaSat03-91, ApaSat29-52, ApaSat30-50, and ApaSat40-189). In addition, the two satDNA families (ApaSat02-236 and ApaSat04-233) observed as centromeric sequences, each demonstrated a large deletion in the *Astyanax* species, as well as different abundances in the *Psalidodon* and different mutations fixed on the species of this group.

### Diversification of Abundant SatDNA in *Psalidodon*: New Variants Observed in *Psalidodon*

**FIGURE 2:**
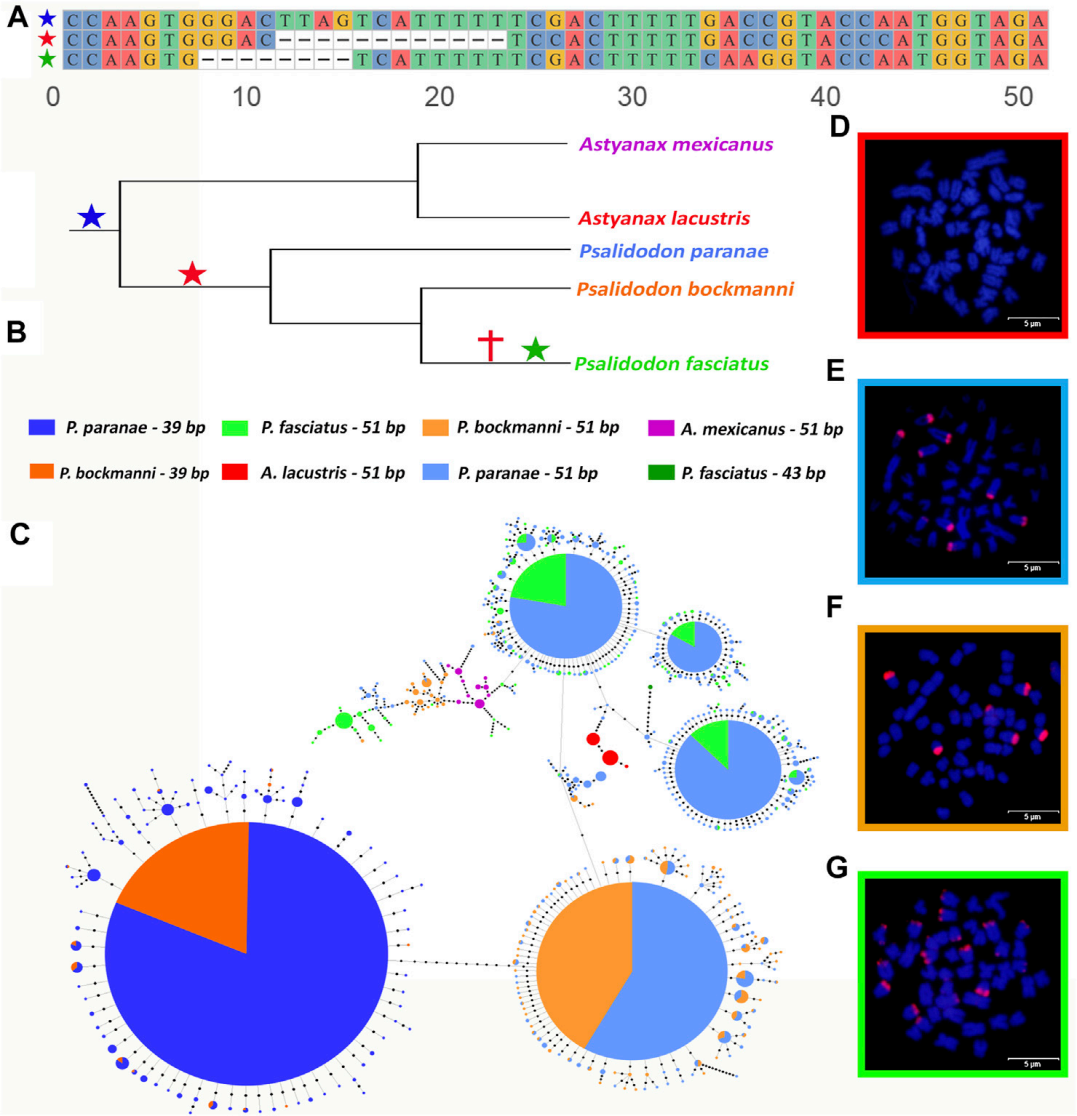
Structure and conservation of As51 monomers between *Psalidodon* and *Astyanax.* Alignment of As51 variants, with 51 bp, 39 bp, and 43 bp, respectively **(A)**. The emergence and disappearance of each variant is demonstrated in the phylogeny of species (blue = 51 bp, red = 39 bp, green = 43 bp) **(B)**. Stars represents the surging of a variation of As51 and crosses represents the elimination of a variation of As51. Linear MST demonstrating the haplotypes of variants of As51 in *Astyanax* and *Psalidodon* species **(C)**. Highlight of the dominance of a haplotype of As51-39 bp shared between *Psalidodon bockmanni* and *Psalidodon paranae*, and the most relevant of four haplotypes of As51-51 bp shared between *P. bockmanni* and *P. paranae* (1) and *P. paranae* and *Psalidodon fasciatus* (3) **(C)**. As51 do not demonstrates FISH signals in *Astyanax lacustris* **(D)**, but this marker forms greats cluster on chromosomes of *P. bockmanni*, *P. paranae*, and *P. fasciatus* **(E–G)**. Fish images were captured in a magnification of ×1000.

The As51 satDNA family was present in the satellitomes of *P. paranae*, *P. fasciatus*, and *P. bockmanni*, corresponding to the most abundant satDNA in these three species. However, this sequence is part of the superfamilies of *P. fasciatus* and *P. bockmanni*. New variants in these two species were produced mainly by deletion of parts of the original sequence, resulting in variants of 39 bp (PboSat03-39) and 43 bp (Pfasat55-43) (Figure 2). We produced a minimum spanning tree (MST) of the As51 satDNA and its variants using monomers extracted from *P. paranae*, *P. fasciatus*, *P. bockmanni*, *A. lacustris*, and *A. mexicanus*, excluding the sequence variants found only once (singletons) (Figure 2). The MST of the 39 bp variant was restricted to *P. bockmanni* and *P. paranae*, although this sequence was missing from the *P. paranae* satellitome, with several haplotypes shared between these two species, including the most abundant. The variant of 43 bp was restricted to *P. fasciatus*, with low abundance. None of the As51 monomers were shared among more than two species, and most only between *P. paranae* and *P. bockmanni* or *P. paranae* and *P. fasciatus*, corroborating the phylogeny of the group. Monomers of As51 were isolated in the genomes of *A. lacustris* and *A. mexicanus*, despite the absence of these satDNAs in the satellitomes of these species and the absence of FISH signals on their chromosomes.

### Cytogenetic Mapping of Conserved Satellite DNA Families in *Astyanax* and *Psalidodon*

**FIGURE 3:**
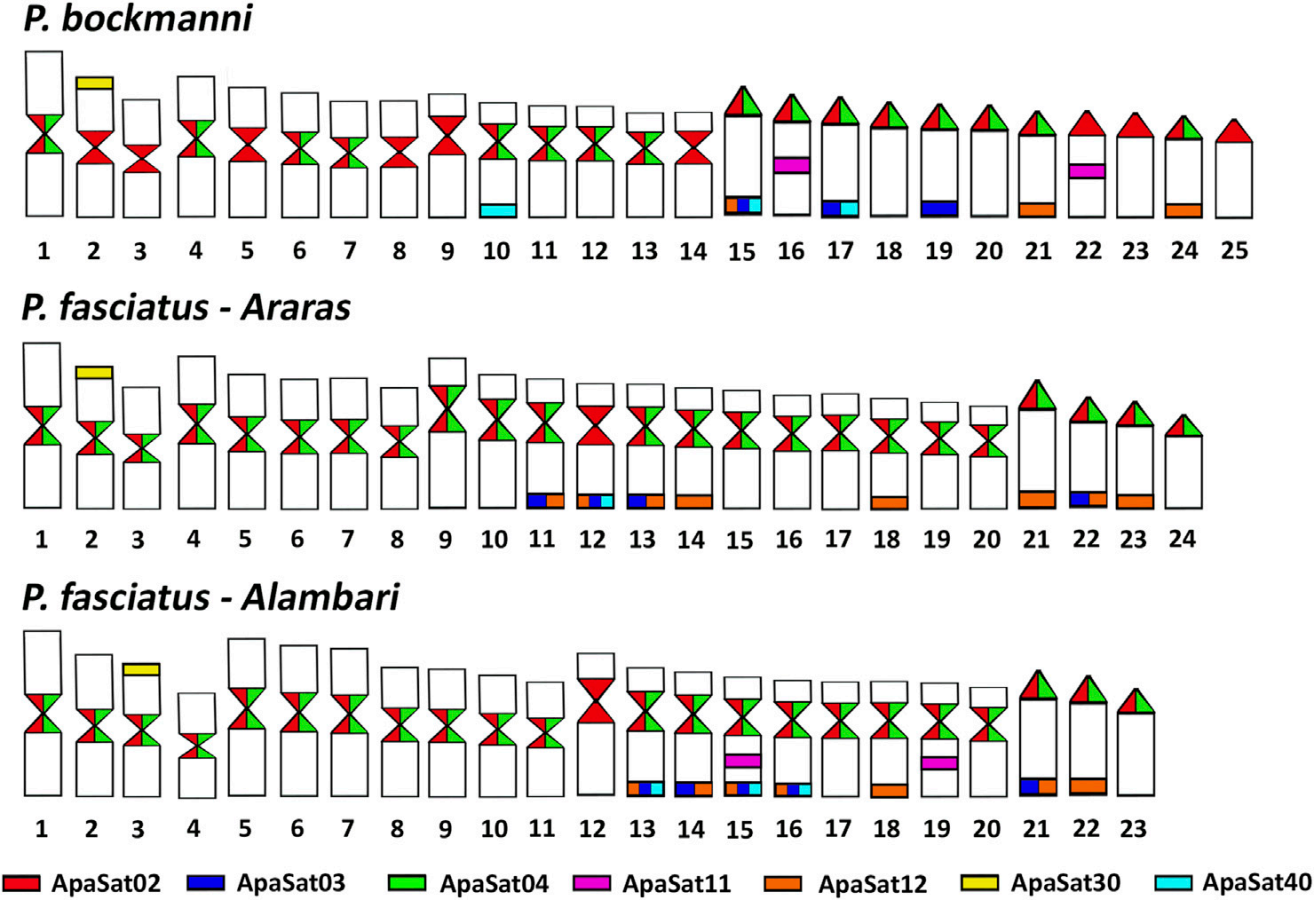
Ideogram of clustered conserved satDNAs in *Psalidodon bockmanni* and two populations of *Psalidodon fasciatus*. No signal was observed in *Astyanax lacustris*.

We performed cytogenetic mapping of eight of the conserved satDNA families in the three species (Figure 3), except for the telomeric sequence (Apasat07-6-tel) and Apasat08-35, in which PCR amplification failed. We utilized the metaphase plates of *A. lacustris*, *P. bockmanni*, and two cytotypes of *P. fasciatus*. None of the satDNA families analyzed here demonstrated clusters on the chromosomes of *A. lacustris*, so we considered them as non-clustered in this species (Supplementary Figure S11). Additionally, Apasat29-52 did not cluster with any individual in our analysis. Clustered satDNAs were mainly present in heterochromatic subtelomeric and centromeric areas. All species demonstrated the same pattern of clusterization of satDNAs, but the number of chromosomes with cluster signals varied. We highlight the following: 1—Apasat02-236 and Apasat04-233 were clustered in pericentromeric regions, with Apasat02-236 present in all chromosomes of *P. fasciatus* and *P. bockmanni*, and Apasat04-233 in approximately half of the chromosomes of *P. bockmanni* and absent only on in par 12 in *P. fasciatus;* 2—Apasat30-50 demonstrated conserved clustered positions on the short arms of a pair of metacentric chromosomes in *P. bockmanni* and *P. fasciatus*; and 3—all other satDNAs demonstrated clusters in subtelomeric regions, with the exception of Apasat11-22 that had clusters in the interstitial regions of a pair of acrocentric chromosomes in *P. bockmanni* and two pairs of subtelocentric chromosomes in *P. fasciatus*.

## Discussion

In this study, we performed, for the first time for Neotropical fishes, an evolutionary comparison of the complete satellitome in four species. We observed a high retention of satDNAs in *Psalidodon* and *Astyanax*, demonstrated by the low number of species-exclusive satDNAs (8 for *P. bockmanni*, 15 for *P. fasciatus*, six for *A. lacustris,* and 16 for *P. paranae*). According to the library hypothesis (Fry and Salser, 1977), a group of related species should share a common library of satDNAs, and satellitomes can demonstrate quantitative differences among species due to differential amplification. Therefore, in addition to the possibility that these species-specific satDNAs have appeared *de novo*, future studies could find monomers of these sequences in low abundance on the genome of the other correlated species. A high degree of satDNA families found in the four satellitomes analyzed were shared between the species (Tables 5, 6), supporting the existence of a common library. However, divergences in the abundance of correlated satDNA families were found, as predicted by the library hypothesis (Fry and Salser, 1977). As an example, Alasat07-189 was correlated with Apasat40-189. Changes in satDNA abundance can occur by unequal crossing-over (Garrido-Ramos, 2017), replication slippage (Walsh, 1987; Stephan, 1989; Ruiz-Ruano et al., 2018), replication of extrachromosomal circles of tandem repeats by rolling-circle replication (Cohen et al., 2005; 2010), and transposition element actions (Jurka et al., 2005; Šatović and Plohl, 2013). Comparative analyses of satellitomes of correlated species also found a high degree of shared satDNA families, as in the grasshoppers *Locusta migratoria* and *Oedaleus decorus* (Camacho et al., 2022), In this case, association between satDNAs families and transposable elements were observed, as LmiSat02-176 and OdeSat17-176 associated with Helitron TEs (Camacho et al., 2022).

The A + T content was the only characteristic with a normal distribution in the satellitomes of *P. bockmanni*, *P. fasciatus*, and *A. lacustris*, similar to that found in the satellitomes of *P. paranae* (Silva et al., 2017) and *Megaleporinus macrocephalus* (Utsunomia et al., 2019). However, no correlations were observed between A + T content and RUL, as identified in *P. paranae*, or divergence and abundance, as identified in *M. macrocephalus*.

In addition to the Charsat01-52 and telomeric sequence, we found another eight satDNA families present on the four satellitomes. These satDNAs were maintained from 11.2 mya, when *A. lacustris* diverged from *Psalidodon* (Piscor et al., 2019). The maintenance of satDNA families across different species can occur through the biological function of determinate satDNA (Fry and Salser, 1977) or independence of natural selection (Stephan, 1986; Stephan, 1987; Walsh, 1987; Stephan, 1989; Harding et al., 1992). Despite the occurrence of the transcribed monomers of Charsat01-52 in *P. paranae* (dos Santos et al., 2021), we did not test the transcription of conserved satDNA in our satellitomes. However, the presence of Apasat02-236 and Apasat04-233 in the centromeres of all chromosomes in *Psalidodon* individuals suggests some structural function of these satDNAs. These sequences were related to Alasat08-236, and no FISH signal was observed in *A. lacustris*. Evidence in grasshoppers demonstrated that a satDNA family may be involved in centromeric function in one species, but not in other related species, suggesting that the species had replaced the centromeric satDNA during the evolution process (Camacho et al., 2022). It is common that the more abundant satDNAs are probably involved in centromeric function (Melters et al., 2013), as observed in *P. paranae* (ApaSat02-236 and ApaSat04-233) (Silva et al., 2017); however, in *Eumingus monticola*, the eighth satDNAs in order of decreasing abundance is located only in the pericentromeric regions (Camacho et al., 2022). In addition, examples of species with different satDNAs present in centromeres are common, such as those of chickens (Shang et al., 2010), plants (Iwata et al., 2013), and fishes (*Prochilodus lineatus;* Stornioli et al., 2021).

During the description of the satellitome of *P. paranae* (Silva et al., 2017), those authors obtained FISH signals of *P. paranae* satDNAs from *P. bockmanni* and *P. fasciatus*. Our analyses corroborated their results, with the addition of clustered signals of Apasat12-69 and Apasat40-189 in *P. bockmanni* and *P. fasciatus*. However, FISH signals were not observed in *A. lacustris*. According to the species tree (Silva et al., 2014) *P. paranae* and *P. bockmanni* are closely related species. We observed that approximately 50% of the satDNA families of *P. bockmanni* had some similarity with satDNAs of *P. paranae*, corroborating these results. In addition, the 39 bp variant of As51 (Apasat01-51) was present only in *P. paranae* and *P. bockmanni*.

The As51 satDNA was characterized by digestion of the *KpnI* restriction enzyme in *P. scabripinnis* (Mestriner et al., 2000), and is the most commonly used satDNA cytogenetic marker in this group, with FISH signals in *P. paranae* (Silva et al., 2014), *P. scabripinnis* (Mestriner et al., 2000), *P. fasciatus* (Kantek et al., 2009), and several other species. The description of the satellitome of *P. paranae* revealed that As51 was the most abundant satDNA in this species, corresponding to Apasat01-51. Our results demonstrated that it was the most also abundant in the satellitomes of *P. bockmanni* and *P. fasciatus*, despite its absence in *A. lacustris* satellitome. In addition, variants of this sequence have been described for the first time in *P. fasciatus* (Afasat55-43) and *P. bockmanni* (Abosat03-39, also present in *P. paranae*). Our data suggest the presence of As51 in an ancestor of *Psalidodon* and *Astyanax*, due to the identification of As51-51 monomers in the genomes of *A. lacustris* and *A. mexicanus*, where it remains as a relic in these species, with the absence of other variants in *Astyanax*. Therefore, we suggest the emergence of As51-51 in an ancestor of *Psalidodon*, and *Astyanax* (11.2 mya), with subsequent amplification and diversification of this satDNA in *Psalidodon* resulting in variants of 39 bp (6.5 mya) and 43 bp (2 mya). The absence of a 39 bp variant in *P. fasciatus* may be derived from stochastic processes that have led to significant nucleotide divergence. Similar cases were observed in *Drosophila*, with 1.688 satDNA conserved in a subgroup of species, with the exception of *D. kikkawai* and *D. leontia* (de Lima et al., 2020).

Our results expand the knowledge of the conservation and evolution of satDNAs in *Psalidodon* and *Astyanax*, demonstrating a large degree of sharing of sequences between these genera. In addition, we describe the evolutionary history of As51 with expansion and diversification of this sequence in *Psalidod*o*n*.

## Data Availability

The datasets presented in this study can be found in online repositories. The names of the repository/repositories and accession number(s) can be found in the article/Supplementary Material.
