# Supplementary material for: Revealing the Satellite DNA History in Psalidodon and Astyanax Characid Fish by Comparative Satellitomics
Source: Front Genet. 2022 Jun 21;13:884072. doi: 10.3389/fgene.2022.884072 (PMC9253505; doi:10.3389/fgene.2022.884072)
Supplement: Supplementary file 1 [file DataSheet1.pdf]

Supplementary Material

1 Supplementary Data

1.1 Alignments of conserved satDNAs families between *P. paranae*, *P. fasciatus*, *P. bockmanni* and *A. lacustris*.

PboSat02-235, PboSat04-235, PfaSat02-237, PfaSat23-236, AlaSat08-236, ApaSat02-236, and APaSat04-233

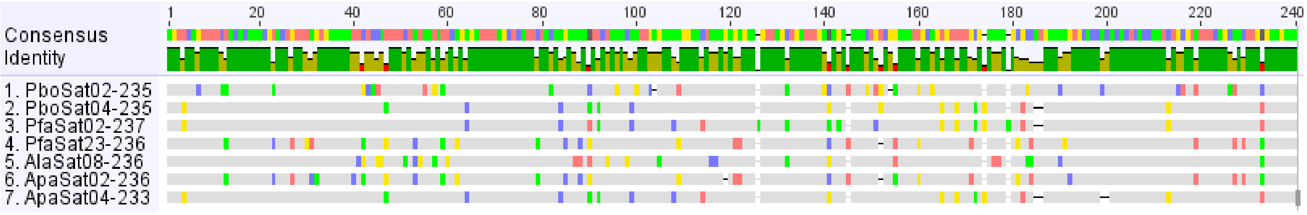

PboSat09-35, PfaSat47-35, AlaSat27-35, and ApaSat08-35

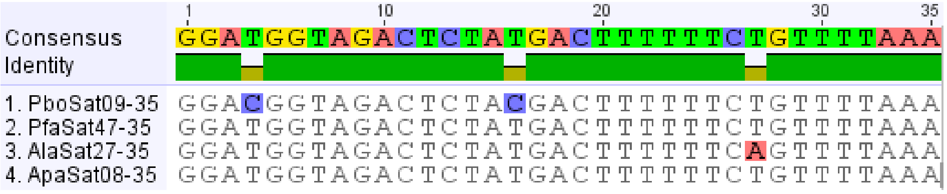

PboSat17-69, PfaSat12-68, AlaSat15-69, and ApaSat12-69

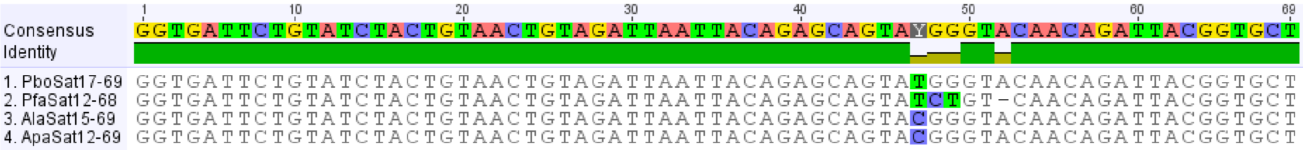

PboSat19-22, PfaSat19-22, AlaSat22-22, AlaSat33-22, and ApaSat11-22

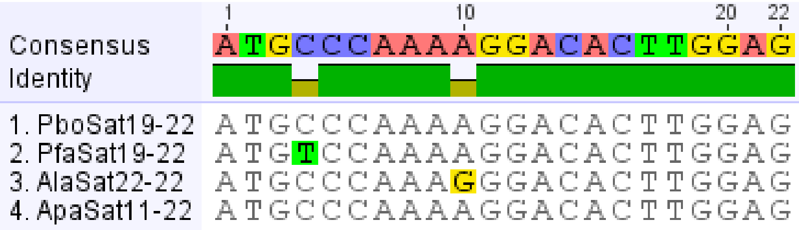

PboSat24-50, PfaSat17-59, PfaSat42-51, AlaSat17-58, and ApaSat30-50

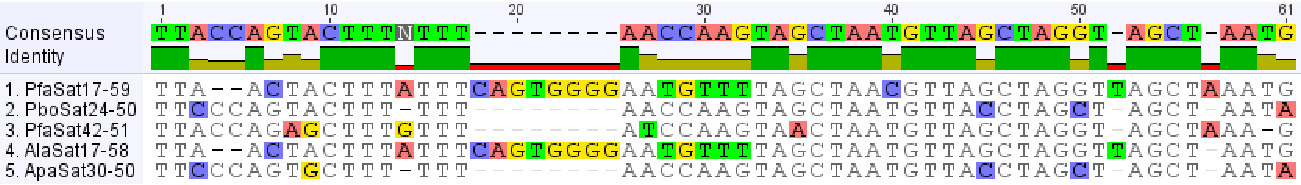

PboSat37-188, PfaSat43-191, AlaSat07-189, and ApaSat40-189

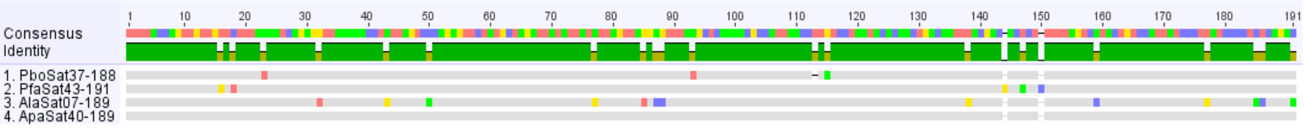

PboSat38-91, PfaSat13-91, AlaSat01-91, and ApaSat03-91

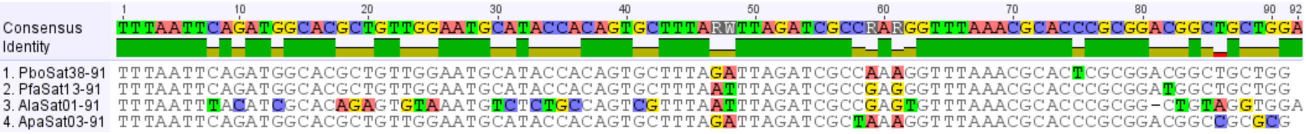

PboSat43-52, PfaSat38-52, AlaSat25-52 and ApaSat29-52. (CharSat01-52)

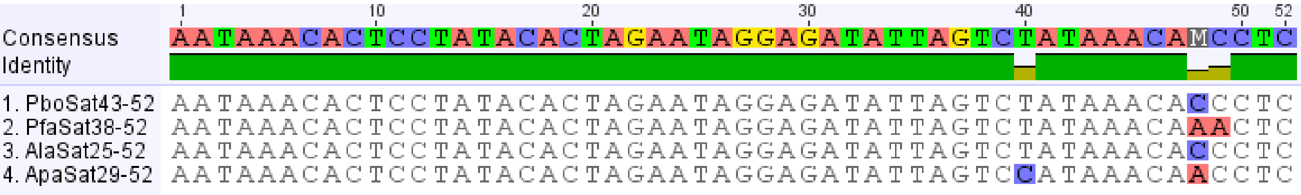

PboSat01-51, PboSat03-39, PfaSat01-51, PfaSat55-43, PfaSat57-51, and ApaSat01-51. (As51)

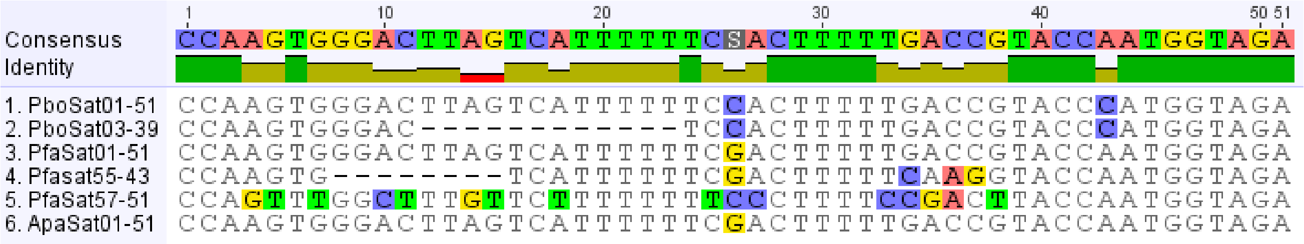

PboSat05-84, PfaSat06-85 and AlaSat10-84.

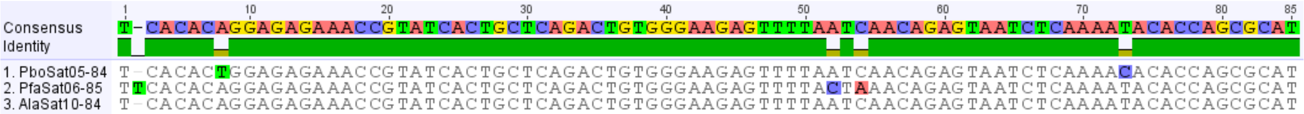

PboSat06-23, PboSat22-22, ApaSat05-23, and ApaSat13-23.

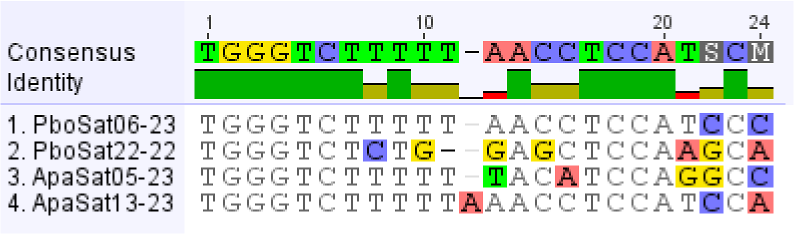

PboSat07-31, PfaSat07-31, and AlaSat09-31.

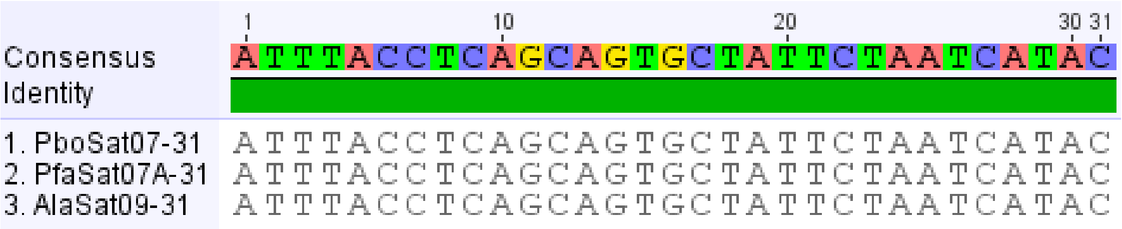

PboSat08-188, PfaSat15-187, and AlaSat32-187.

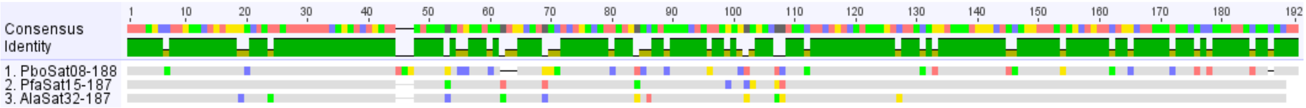

PboSat10-40 and PfaSat14-40.

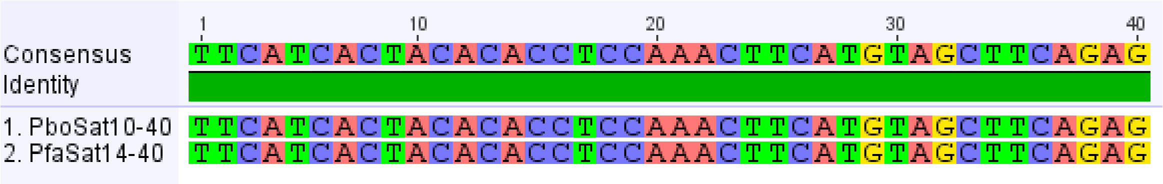

PboSat11-27 and PfaSat18-27

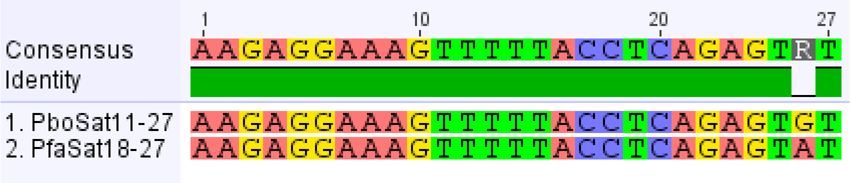

PboSat12-190 and PfaSat29-190.

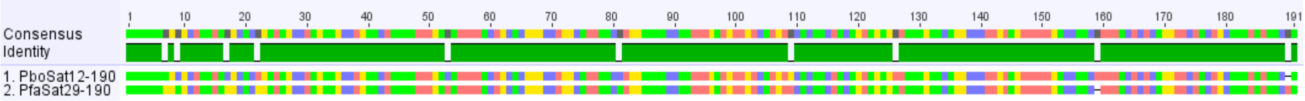

PboSat13-106, PfaSat21-109 and AlaSat19-106.

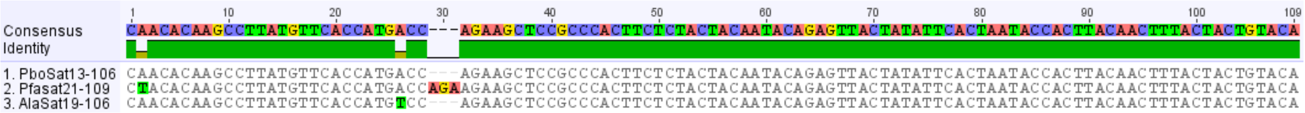

PboSat14-61, PfaSat10-61 and AlaSat14-62.

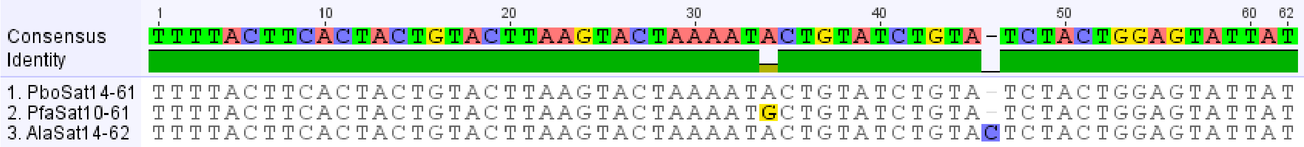

PboSat15-87, Pfa20-76, and AlaSat18-80.

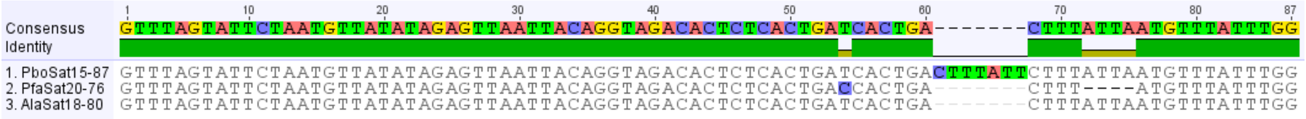

PboSat16-63 and ApaSat19-77

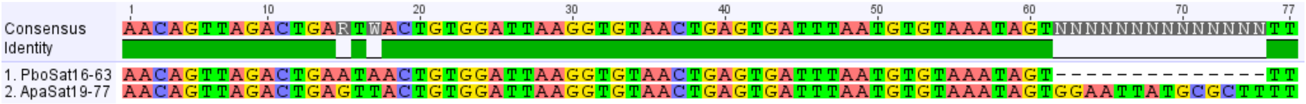

PboSat18-52, PfaSat26-54 and ApaSat16-54

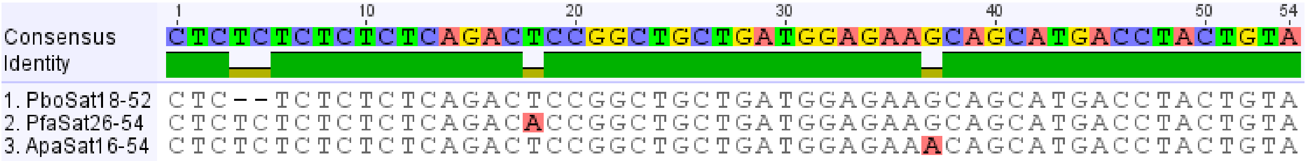

PboSat20-107 and ApaSat38-107

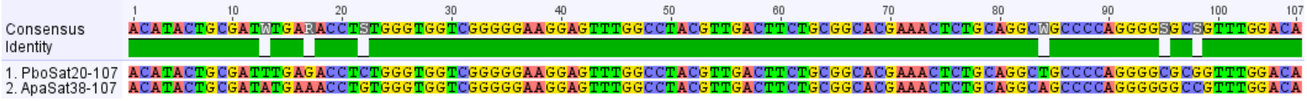

PboSat21-82, PfaSat30-85 and AlaSat20-85

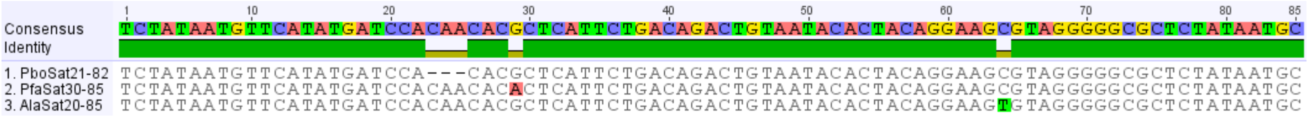

PboSat23-54, PfaSat46-54, and ApaSat18-58

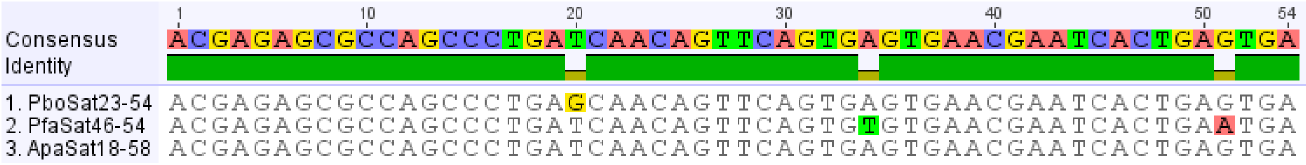

PboSat25-42 and PfaSat31-42

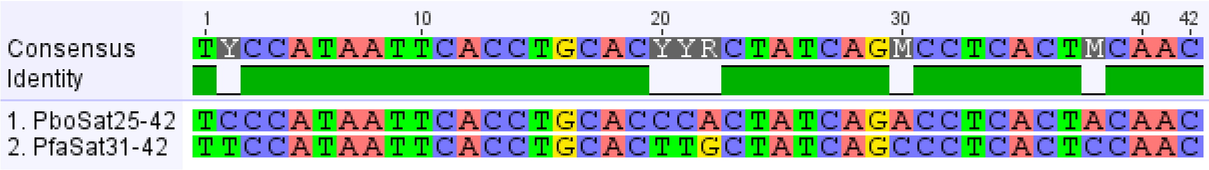

PboSat26-21, PfaSat52-21, and ApaSat36-21

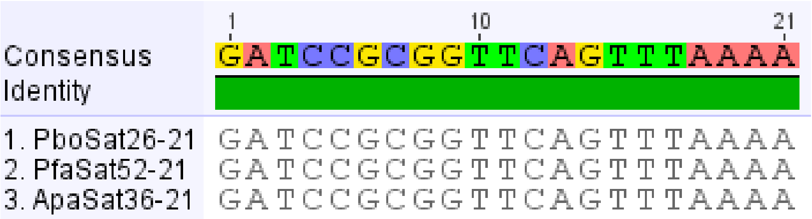

PboSat27-51, PfaSat28-51, and ApaSat15-51

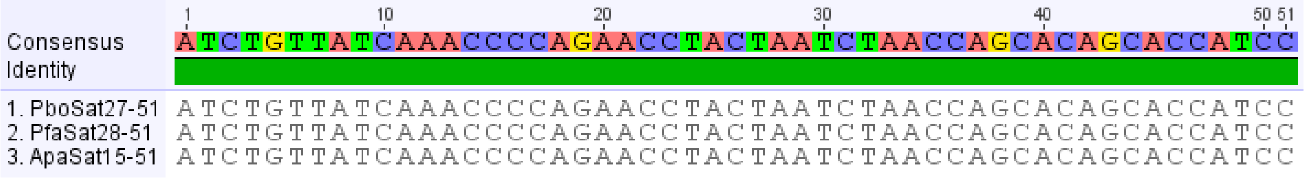

PboSat28-62 and ApaSat22-62

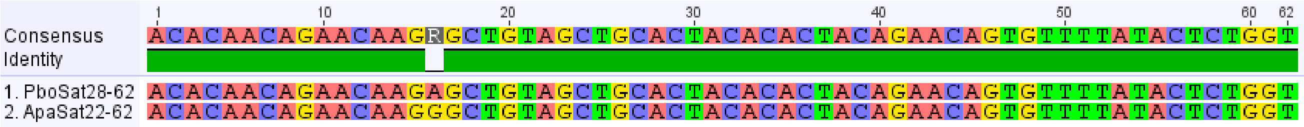

PboSat29-142 and PfaSat40-143

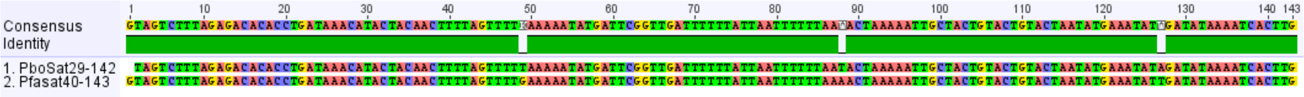

PboSat30-55 and PfaSat56-55

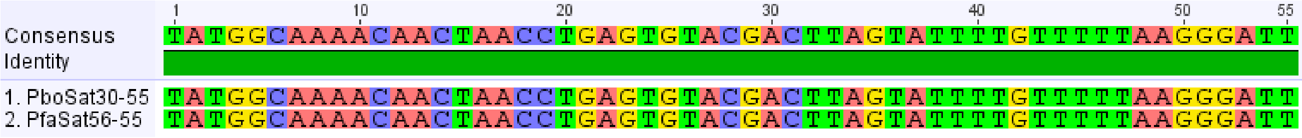

PboSat31-657 and AlaSat31-552

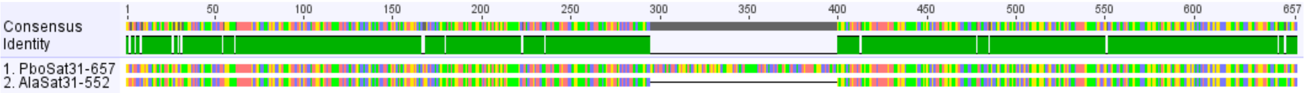

PboSat33-42 and PfaSat49-42

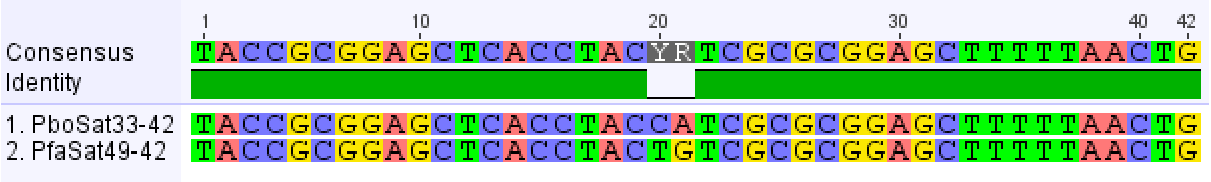

PboSat34-56 and PfaSat54-56

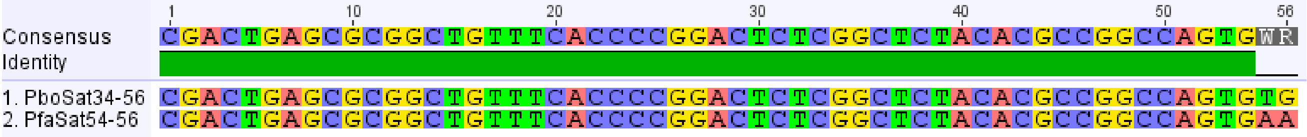

PboSat35-584 and AlaSat24-577

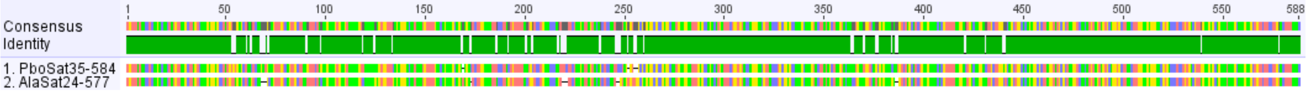

PboSat36-419 and AlaSat26-418

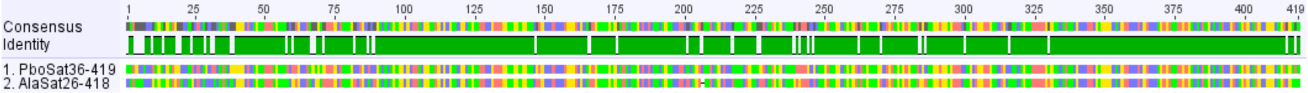

PboSat40-78 and ApaSat24-78

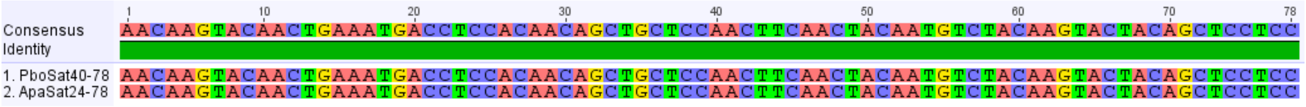

PboSat42-112 and ApaSat33-112

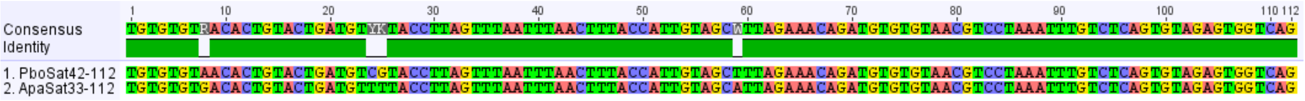

PboSat46-90, PfaSat32-65 and ApaSat42-90

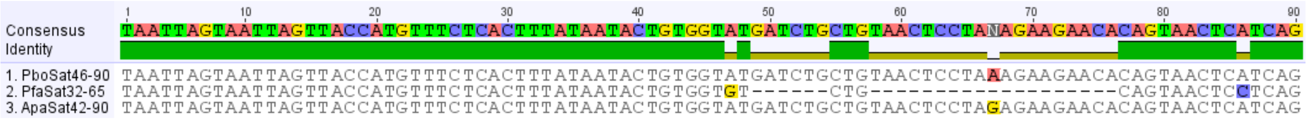

PboSat48-32 and ApaSat39-32

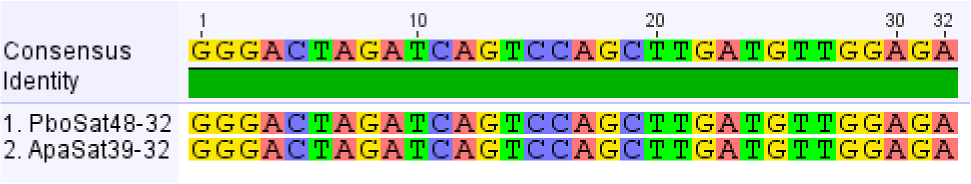

PfaSat08-42 and AlaSat06-42

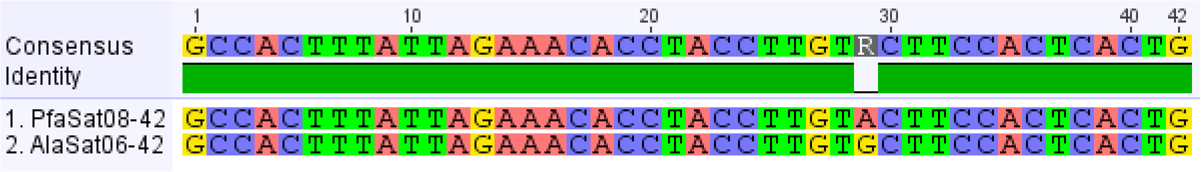

PfaSat09-177 and AlaSat12-177

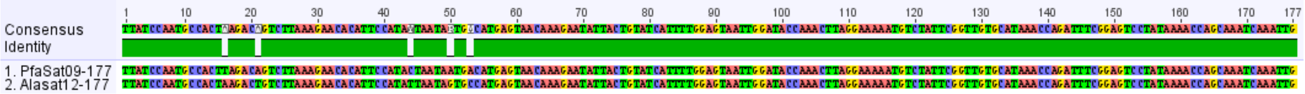

PfaSat11-21 and ApaSat09-21

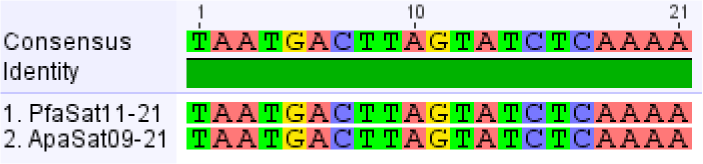

PfaSat36-33, PfaSat22-24 and AlaSat13-24

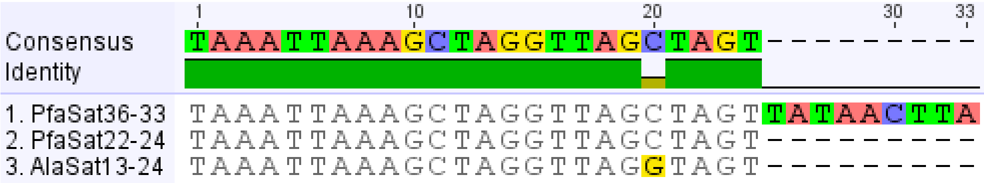

PfaSat24-83 and ApaSat06-86

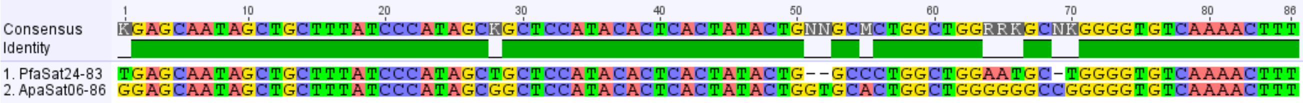

PfaSat27-197 and AlaSat04-151

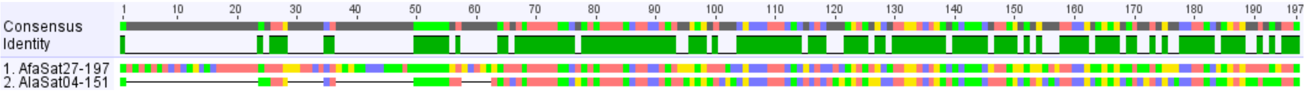

AlaSat02-186 and ApaSat10-179

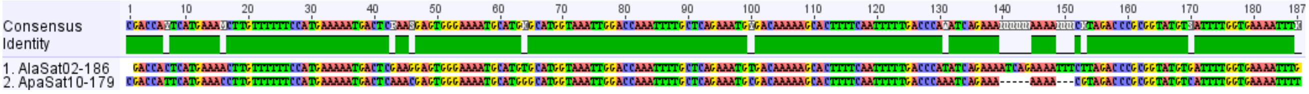

AlaSat05-364 and ApaSat17-365

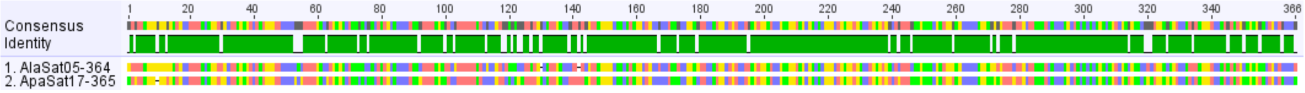

AlaSat29-185 and ApaSat27-178

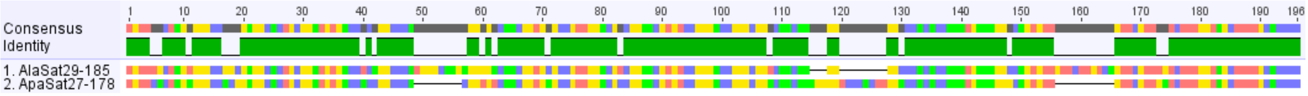

PfaSat01-51, PfaSat55-43 and PfaSat57-51 (SF1)

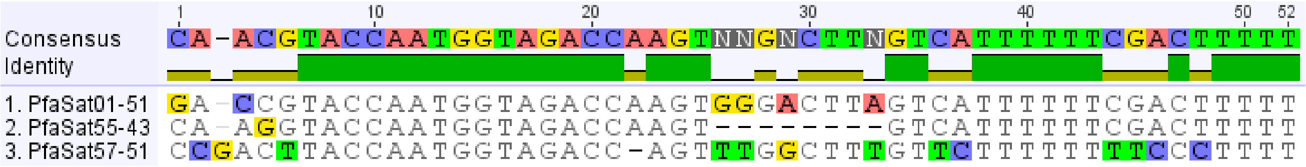

PfaSat02-237 and PfaSat23-236 (SF2)

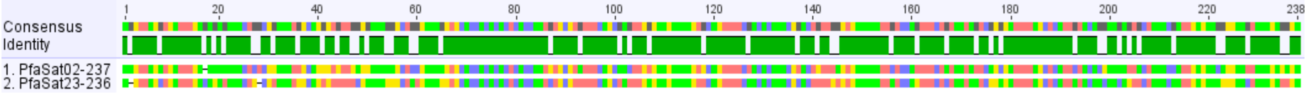

PfaSat17-59 and PfaSat42-51 (SF3)

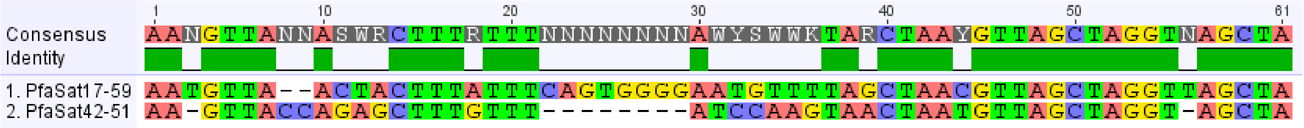

PfaSat22-24 and PfaSat36-33 (SF4)

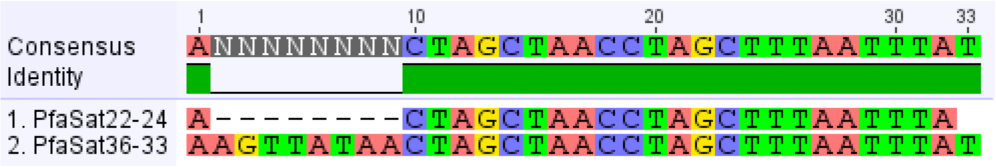

PboSat01-51 and PboSat03-39 (SF1)

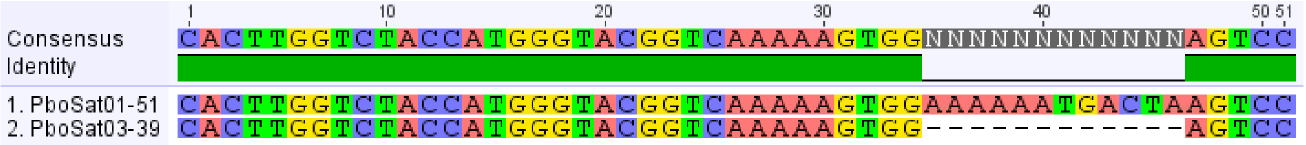

PboSat02-235 and PboSat04-235 (SF2)

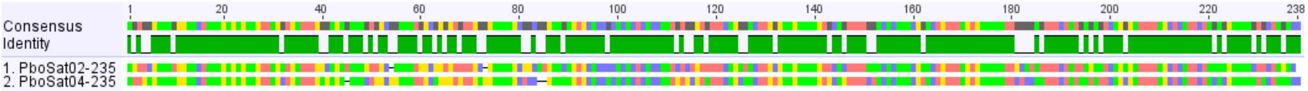

PboSat06-23 and PboSat22-22 (SF3)

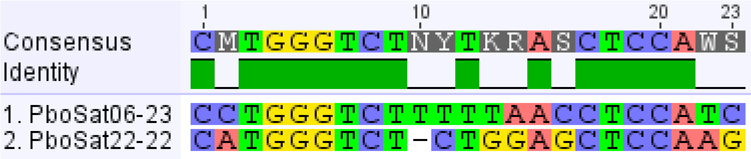

AlaSat22-22 and AlaSat33-22 (SF1)

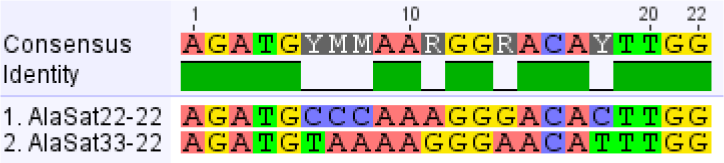

1.2 Repeat profilers of conserved satDNAs families observed on *P. paranae*, *P. bockmanni*, *P. fasciatus*, *A. lacustris* and *A. mexicanus*, respectively

ApaSat02-236

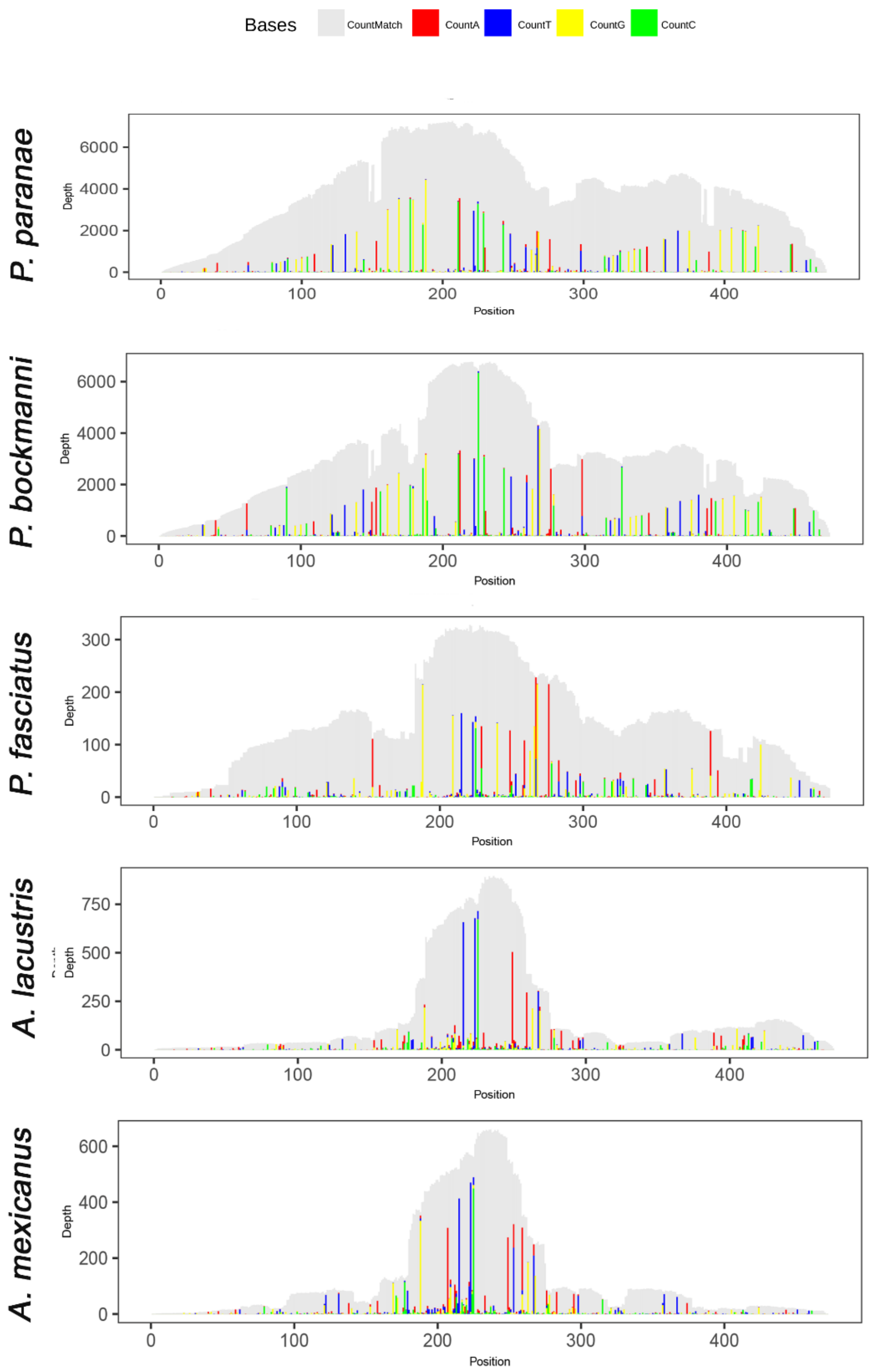

ApaSat03-91

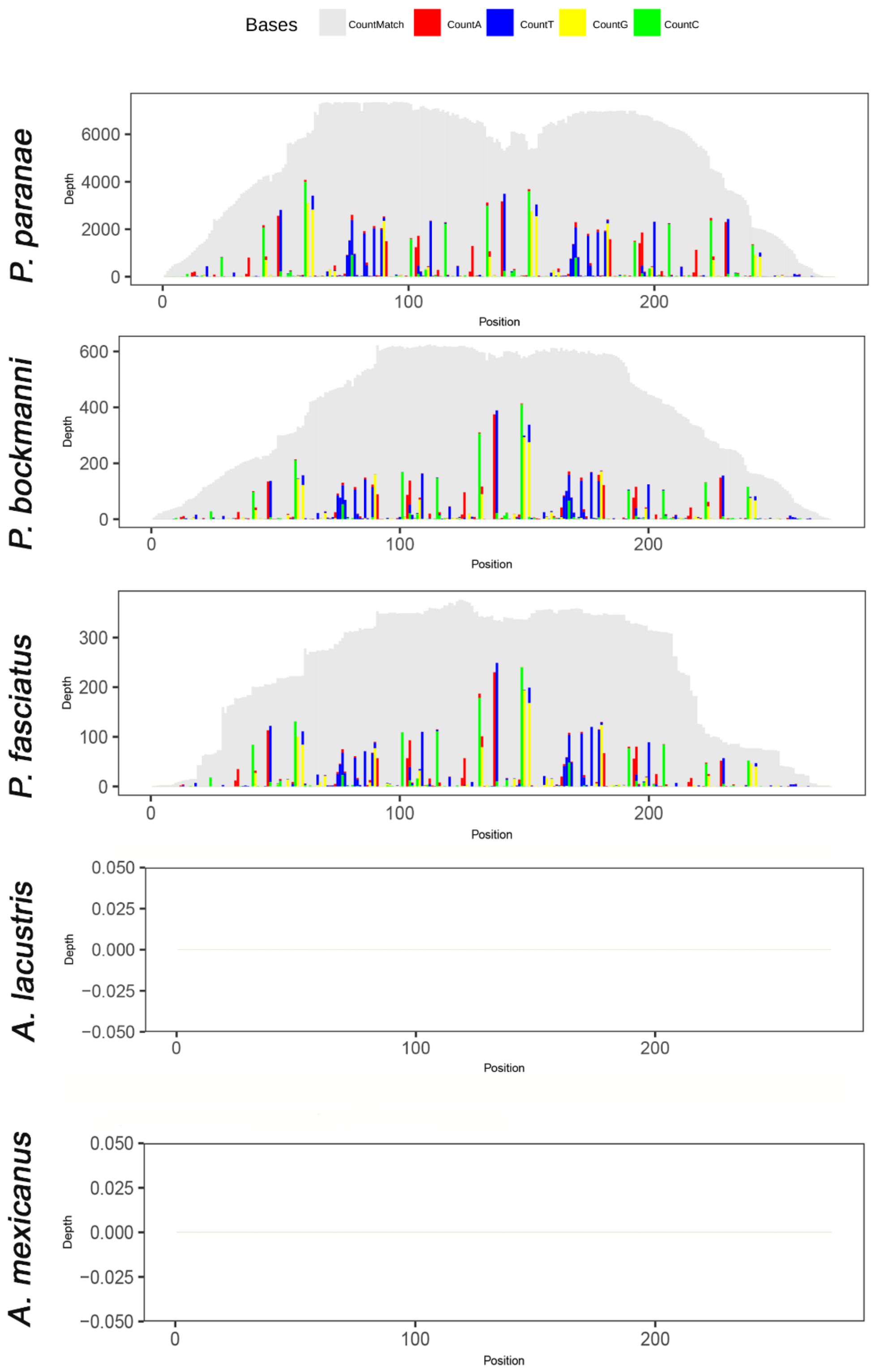

Bases
CountMatch
CountA
CountT
CountG
CountC
P. paranae
P. bockmanni
P. fasciatus
A. lacustris
A. mexicanus
Depth
Position

ApaSat04-233

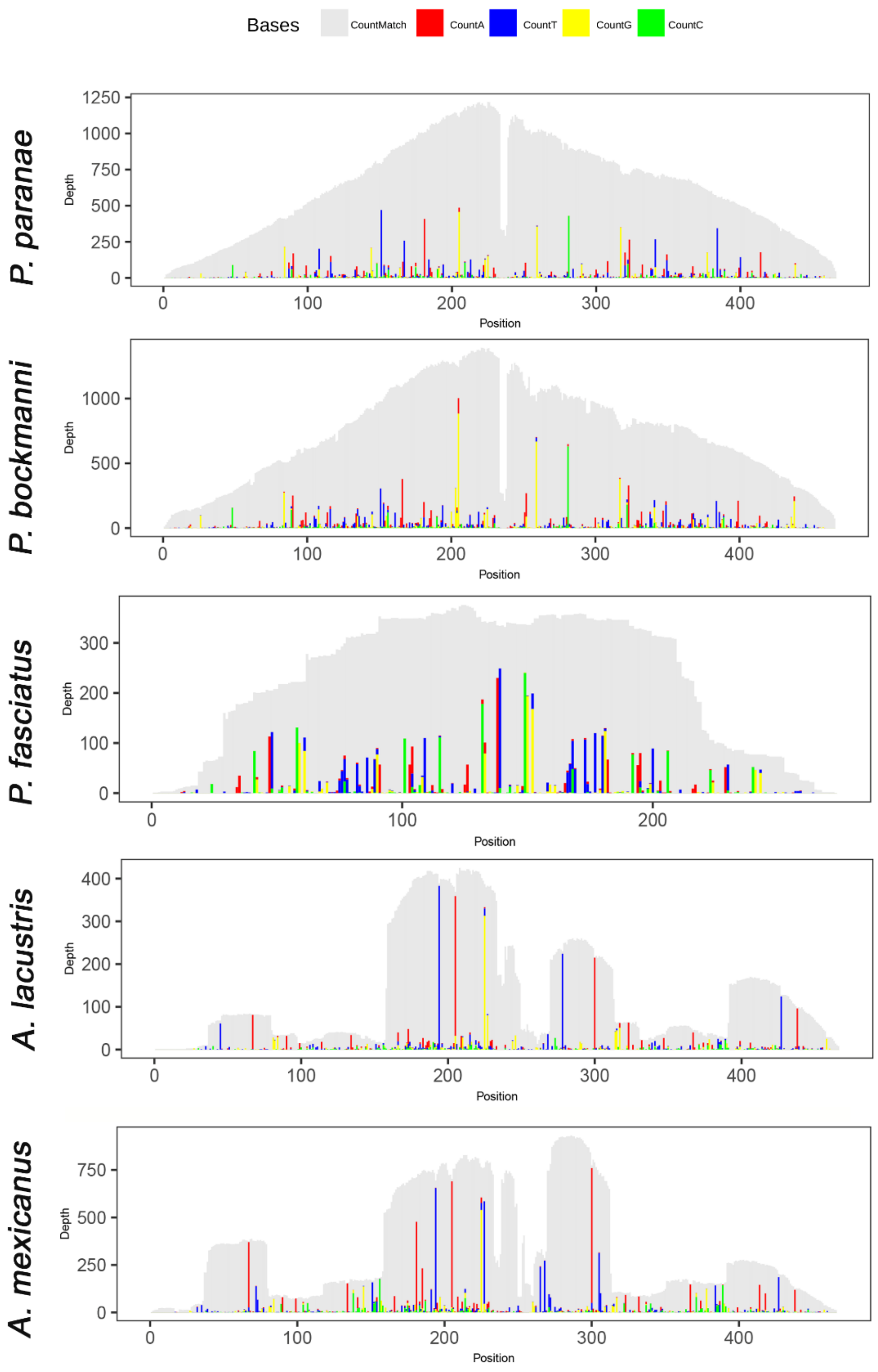

ApaSat07-6-tel

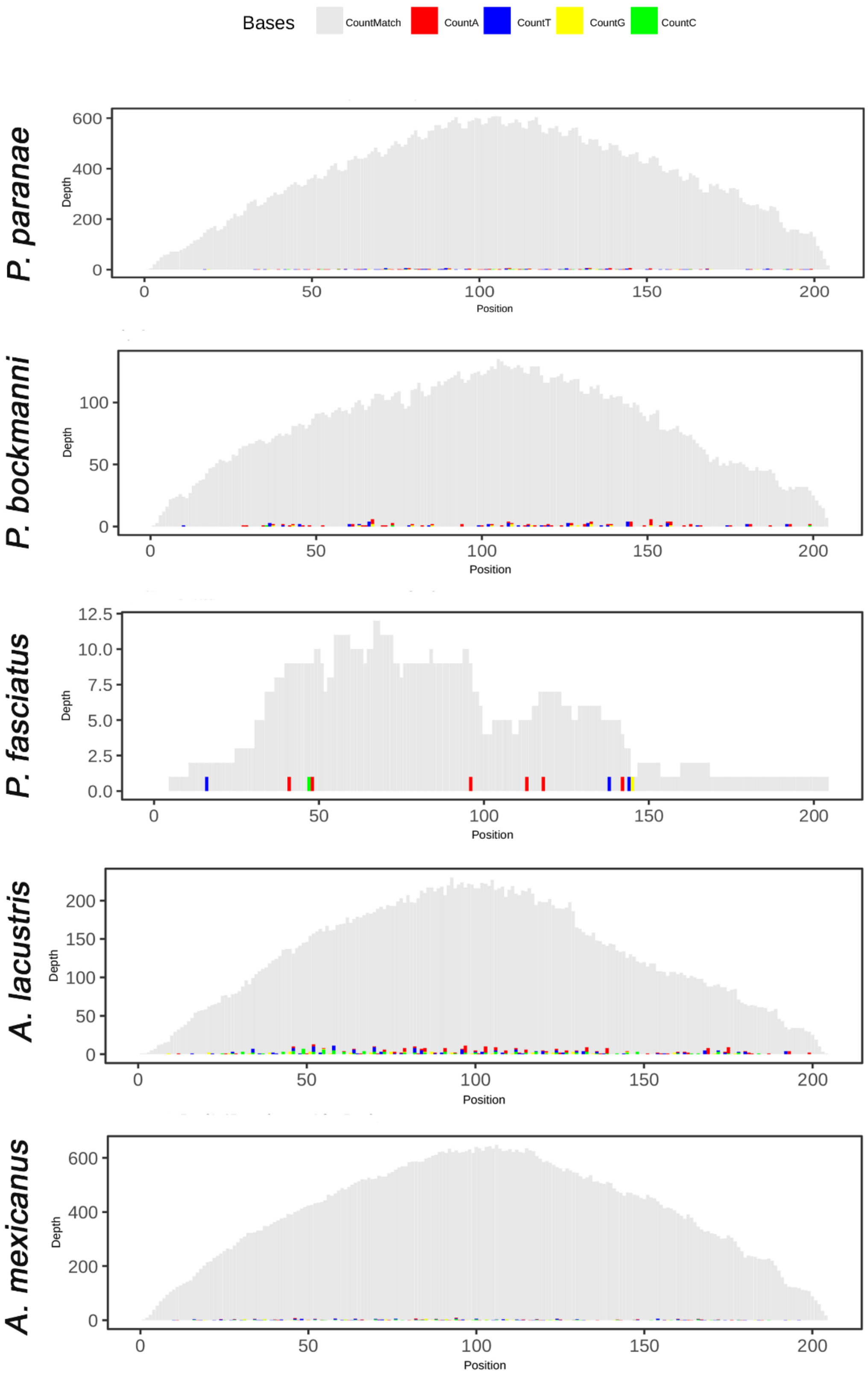

ApaSat08-35

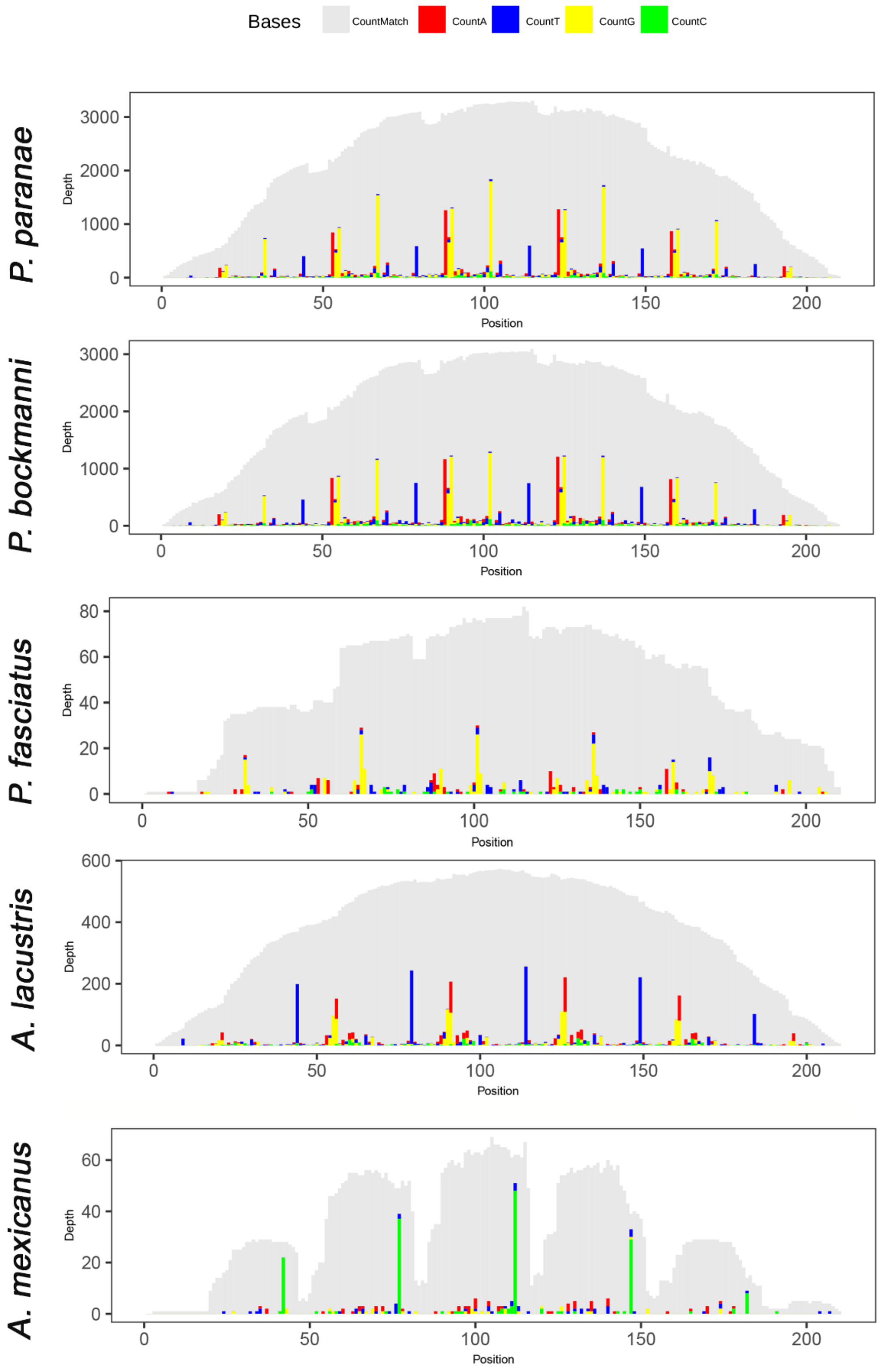
Bases
CountMatch
CountA
CountT
CountG
CountC
P. paranae
P. bockmanni
P. fasciatus
A. lacustris
A. mexicanus
Depth
Position

ApaSat11-22

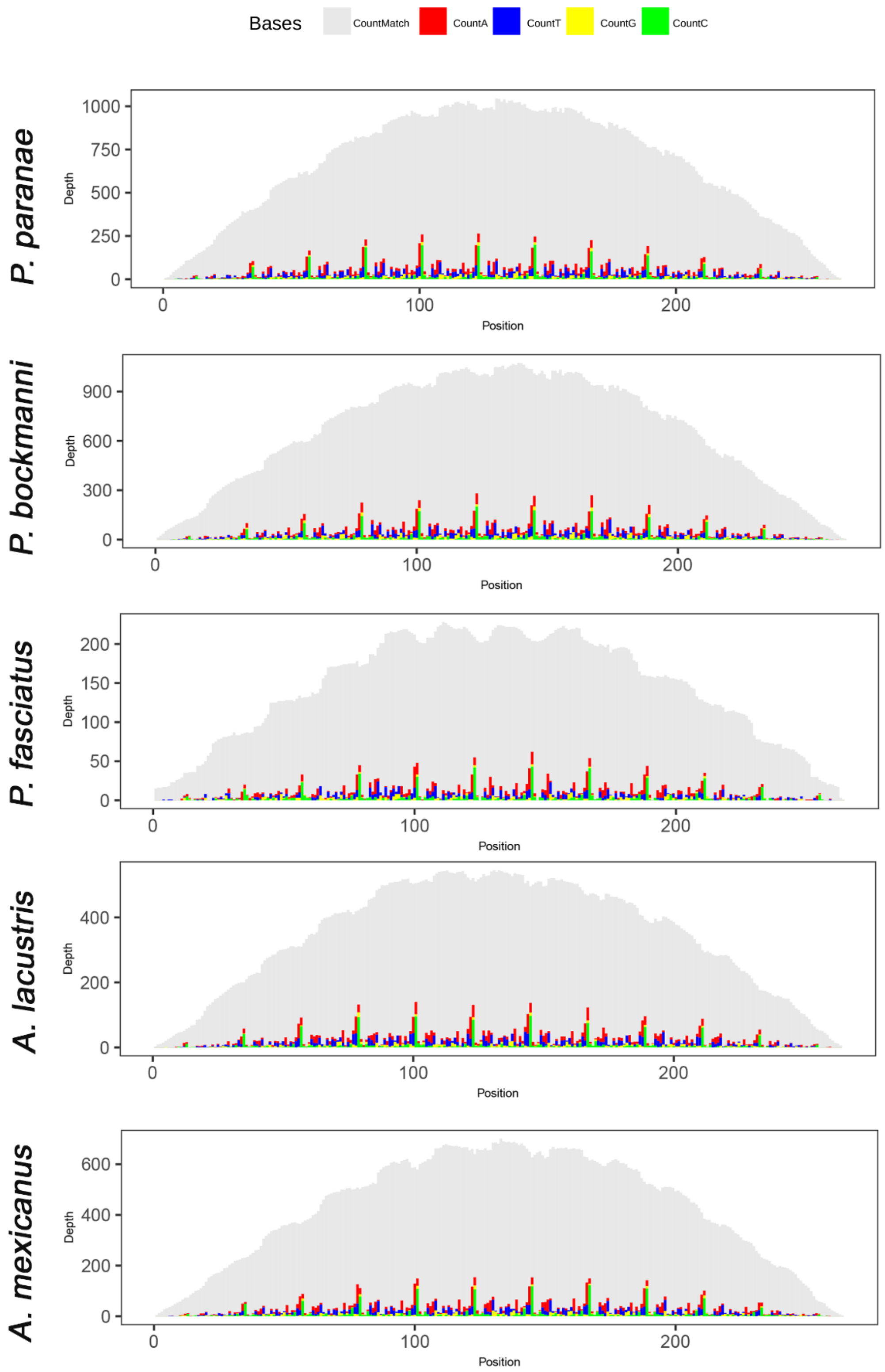

Bases
CountMatch
CountA
CountT
CountG
CountC
P. paranae
P. bockmanni
P. fasciatus
A. lacustris
A. mexicanus
Depth
Position

ApaSat12-69

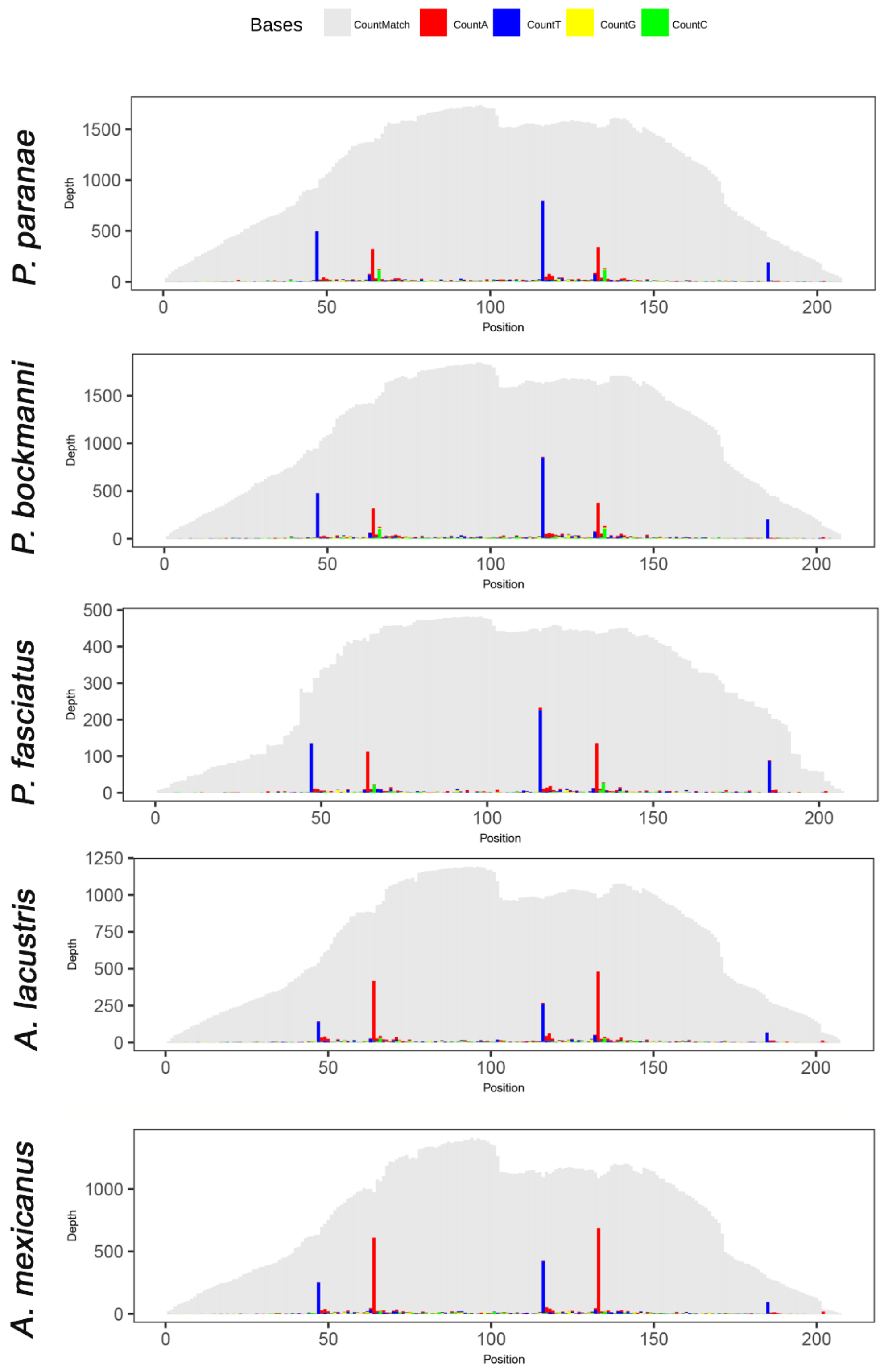

Bases
CountMatch
CountA
CountT
CountG
CountC
P. paranae
P. bockmanni
P. fasciatus
A. lacustris
A. mexicanus
Depth
Position

ApaSat29-52

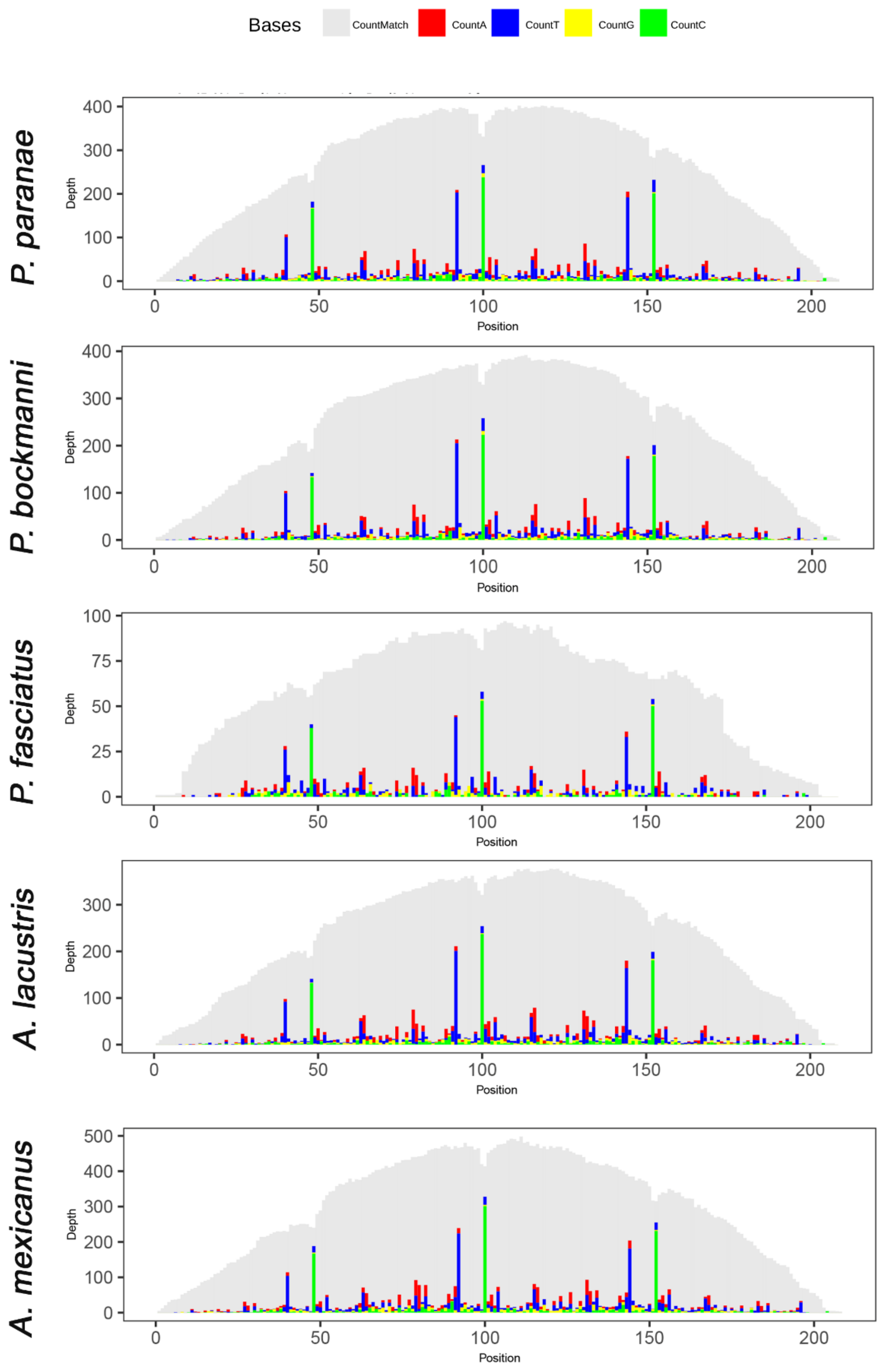
Bases
CountMatch
CountA
CountT
CountG
CountC
P. paranae
P. bockmanni
P. fasciatus
A. lacustris
A. mexicanus
Depth
Position

ApaSat30-50

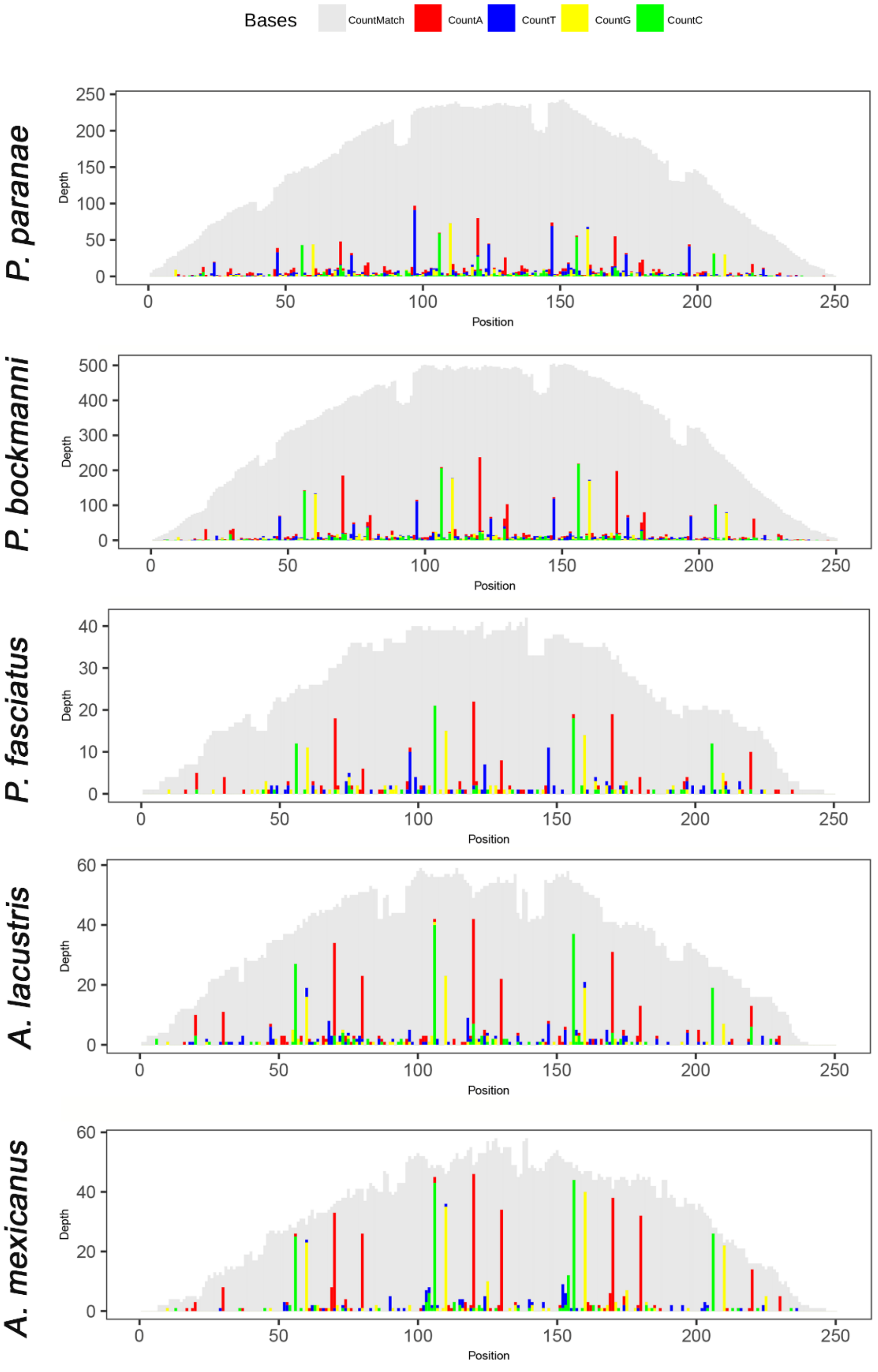
Bases
CountMatch
CountA
CountT
CountG
CountC
P. paranae
P. bockmanni
P. fasciatus
A. lacustris
A. mexicanus
Depth
Position

ApaSat40-189

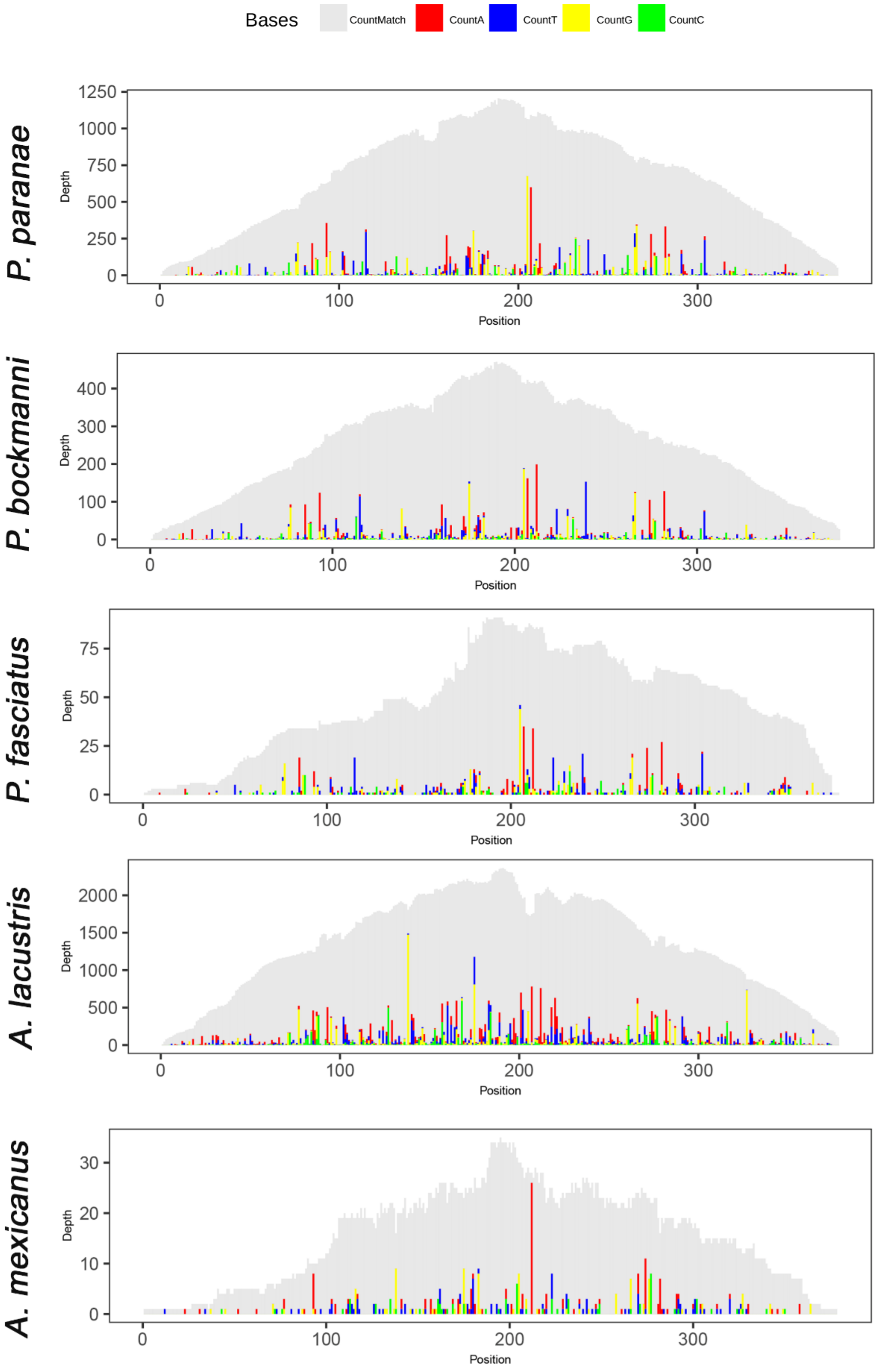

2 Suplementary Tables

Suplementary Table 1: Obtained monomers and utilized monomers of As51 and its variants in *Psalidodon paranae, Psalidodon bockmanni, Psalidodon fasciatus, Astyanax lacustris, and Astyanax mexicanus.* The term "bp" means base pairs.

	**Obtained**		
	**51 bp**	**39 bp**	**43 bp**
P. paranae	16940	8256	0
P. bockmanni	4651	1521	0
P. fasciatus	3570	0	3
A. lacustris	31	0	0
A. mexicanus	36	0	0
	**Utilized**		
	**51 bp**	**39 bp**	**43 bp**
P. paranae	960	474	0
P. bockmanni	260	80	0
P. fasciatus	200	0	3
A. lacustris	31	0	0
A. mexicanus	36	0	0

3 Supplementary Figures

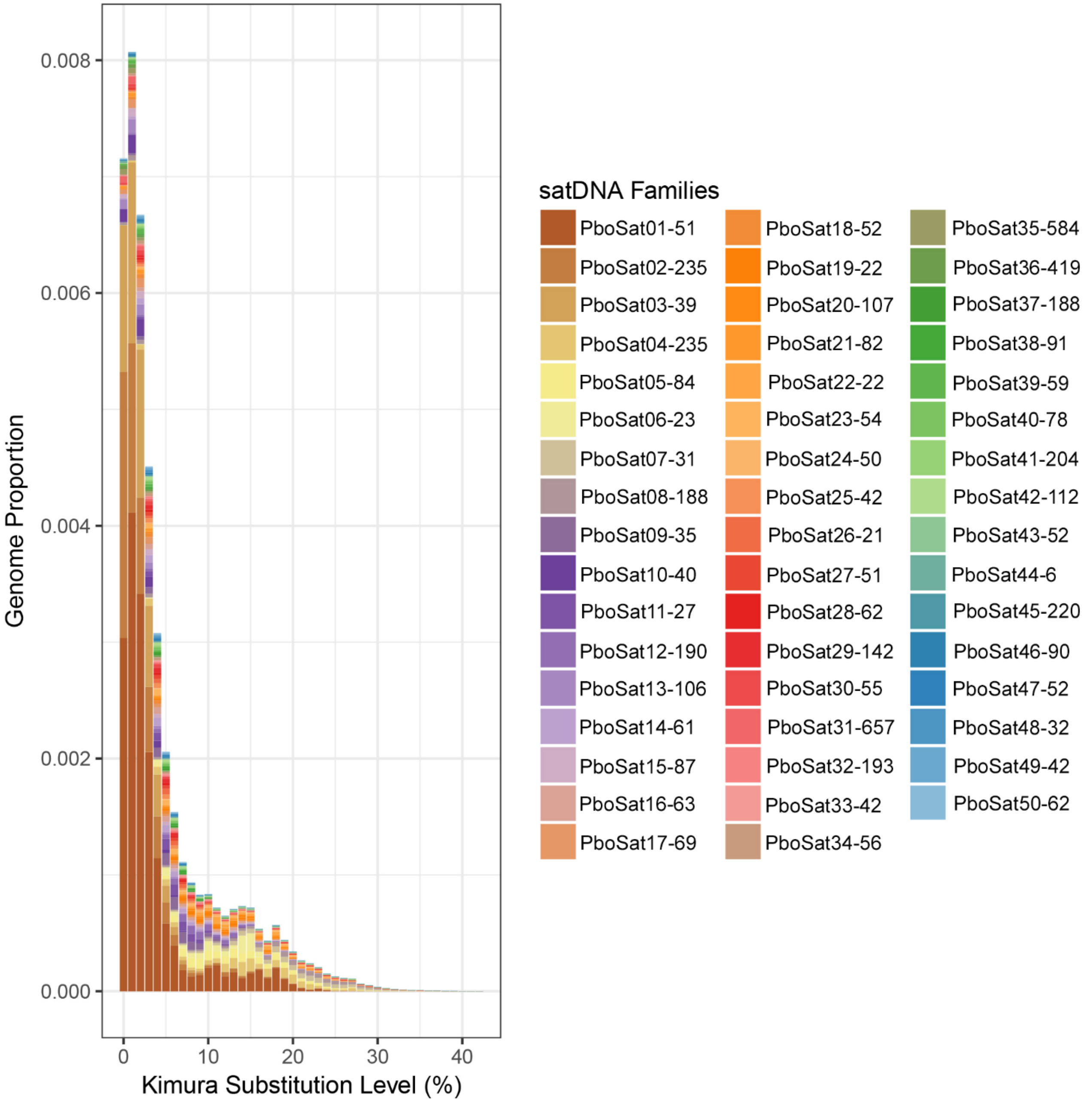

Supplementary Figure 1. Bar color- coded repeat landscapes of total satellitome of *Psalidodon bockmanni.*

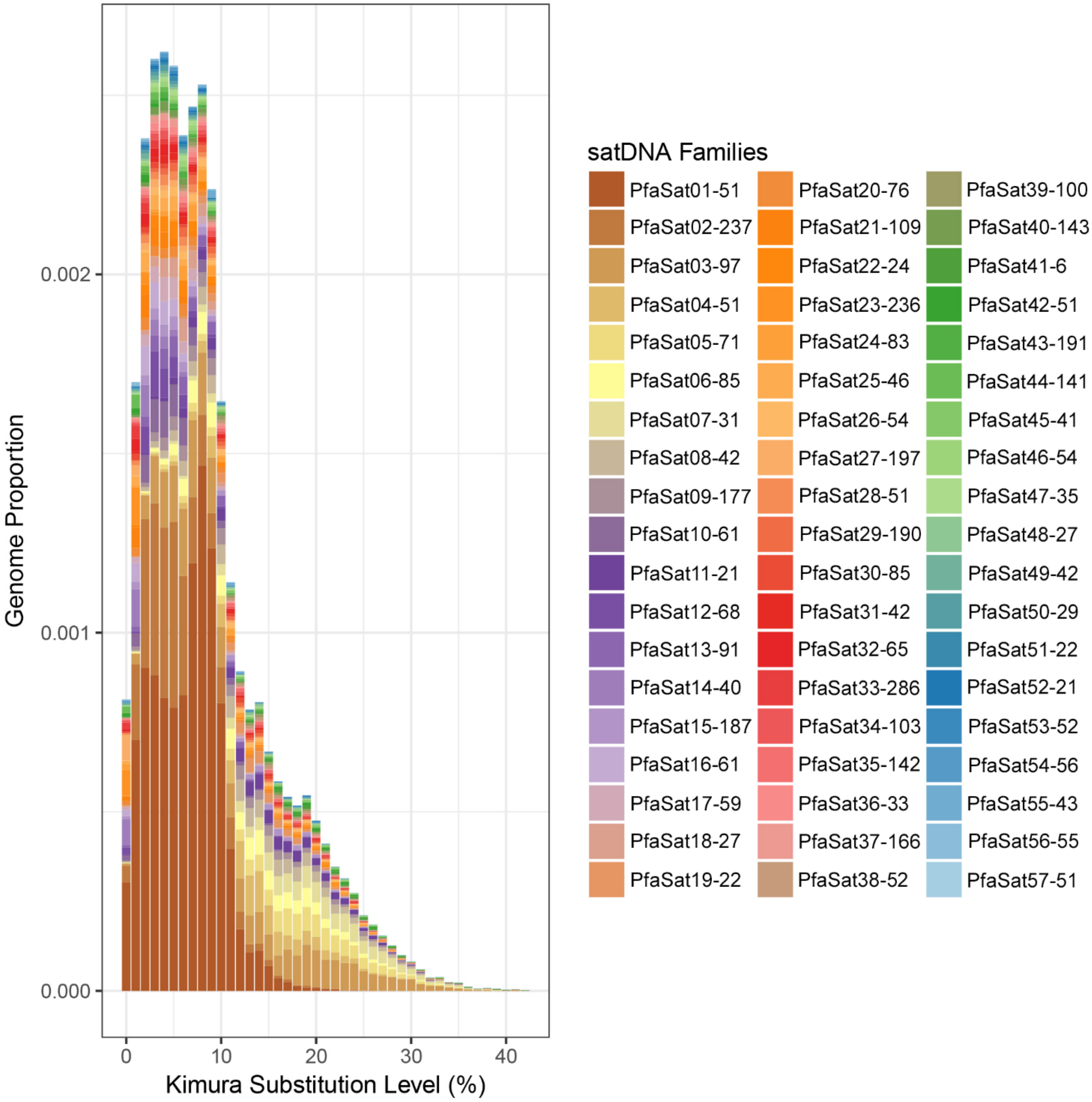

Supplementary Figure 2. Bar color- coded repeat landscapes of total satellitome of *Psalidodon fasciatus.*

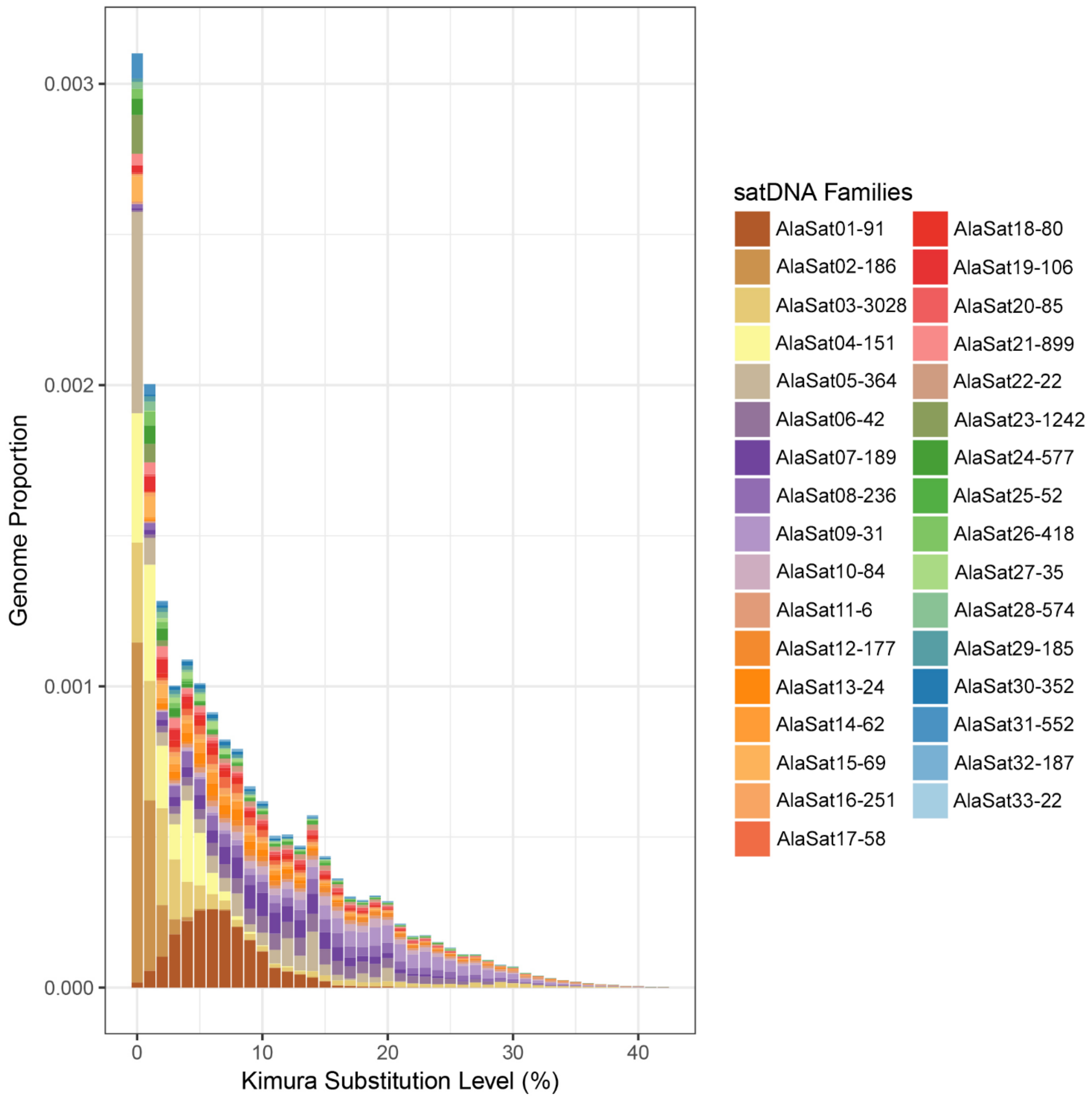

Supplementary Figure 3. Bar color- coded repeat landscapes of total satellitome of *Astyanax lacustris.*

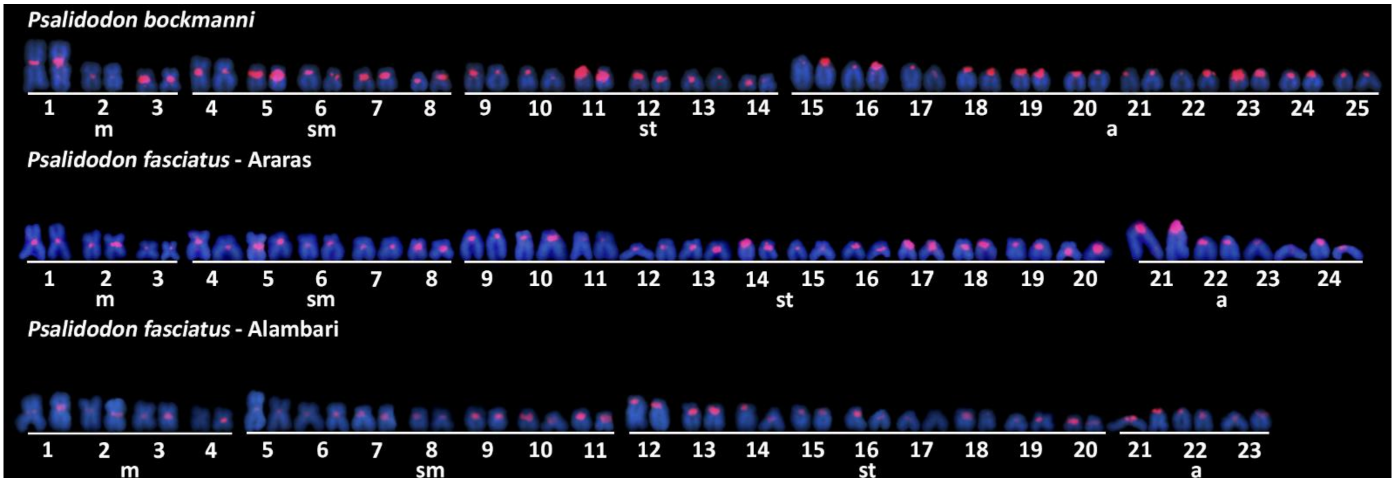

Supplementary Figure 4. Fluorescence *in situ* hybridization (FISH) mapping of ApaSat02-236 in *Psalidodon bockmanni*, *Psalidodon fasciatus* (Araras), and *Psalidodon fasciatus* (Alambari).

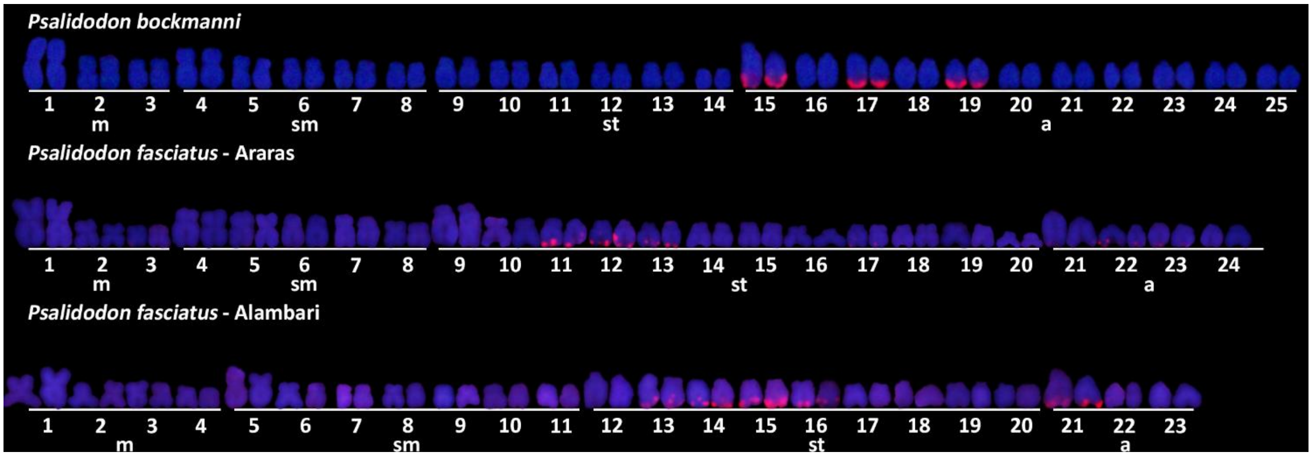

Supplementary Figure 5. Fluorescence *in situ* hybridization (FISH) mapping of ApaSat03-91 in *Psalidodon bockmanni*, *Psalidodon fasciatus* (Araras), and *Psalidodon fasciatus* (Alambari).

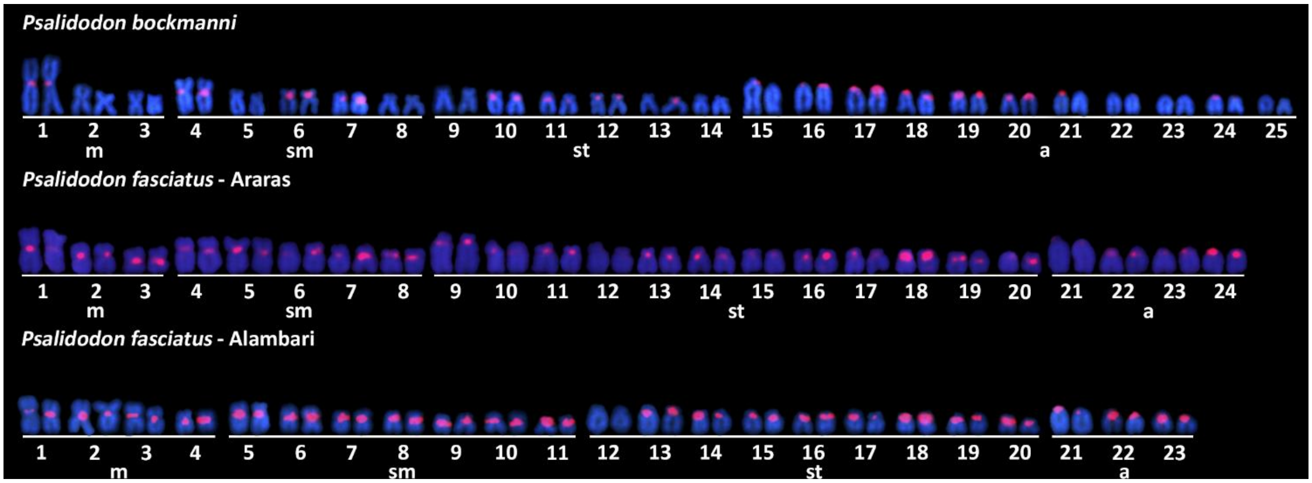

Supplementary Figure 6. Fluorescence *in situ* hybridization (FISH) mapping of ApaSat04-233 in *Psalidodon bockmanni*, *Psalidodon fasciatus* (Araras), and *Psalidodon fasciatus* (Alambari).

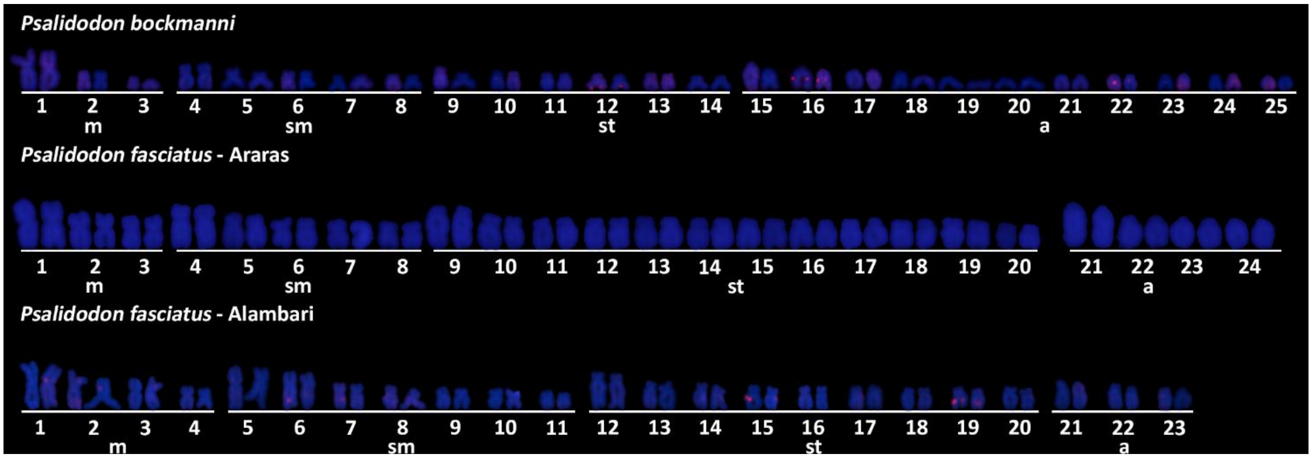

Supplementary Figure 7. Fluorescence *in situ* hybridization (FISH) mapping of ApaSat11-22 in *Psalidodon bockmanni*, *Psalidodon fasciatus* (Araras), and *Psalidodon fasciatus* (Alambari).

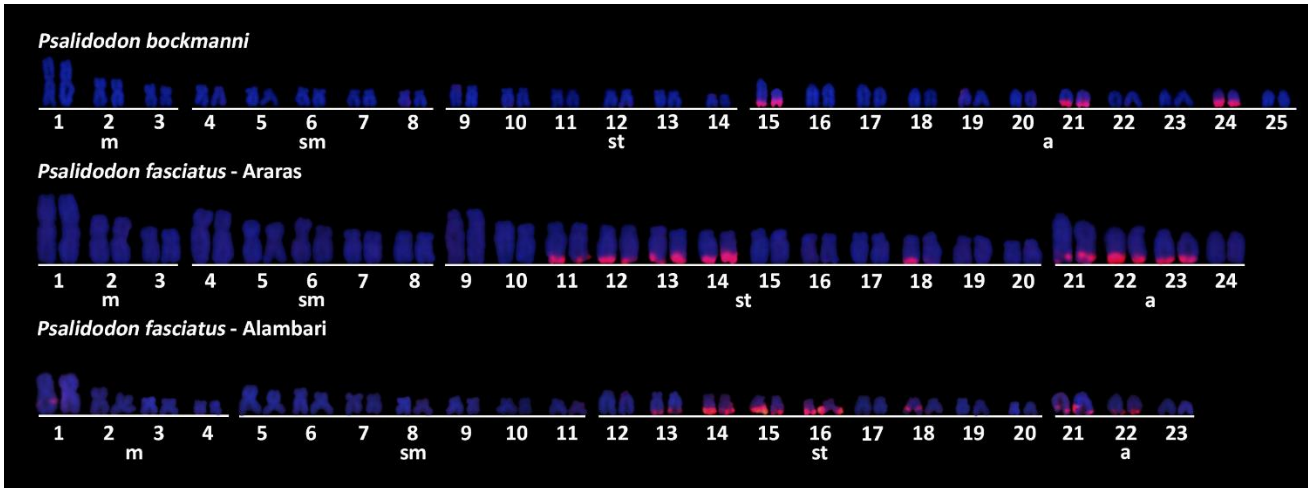

Supplementary Figure 8. Fluorescence *in situ* hybridization (FISH) mapping of ApaSat12-69 in *Psalidodon Bockmanni*, *Psalidodon Fasciatus* (Araras), and *Psalidodon fasciatus* (Alambari).

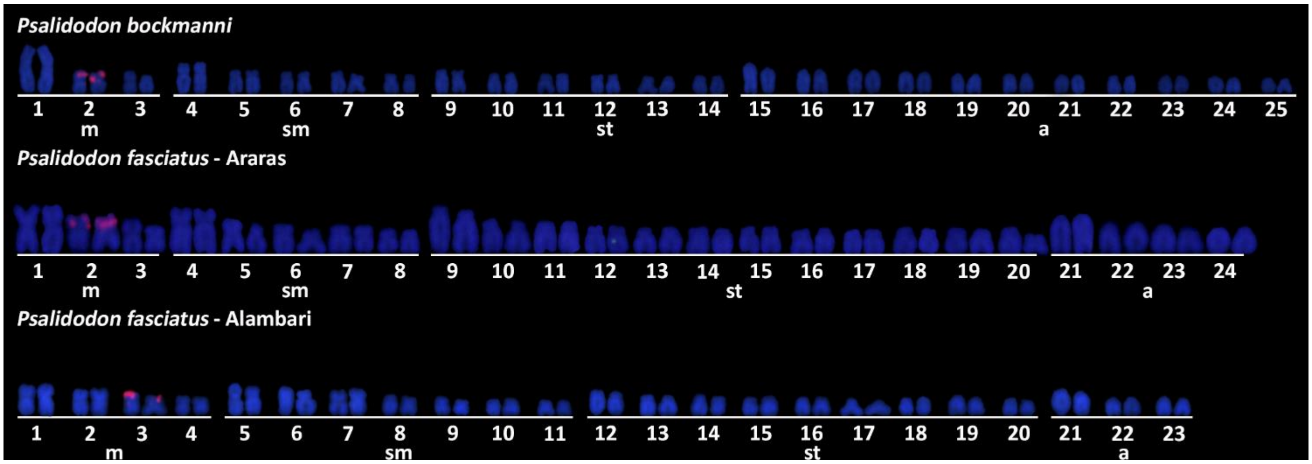

Supplementary Figure 9. Fluorescence *in situ* hybridization (FISH) mapping of ApaSat30-50 in *Psalidodon bockmanni*, *Psalidodon fasciatus* (Araras), and *Psalidodon fasciatus* (Alambari).

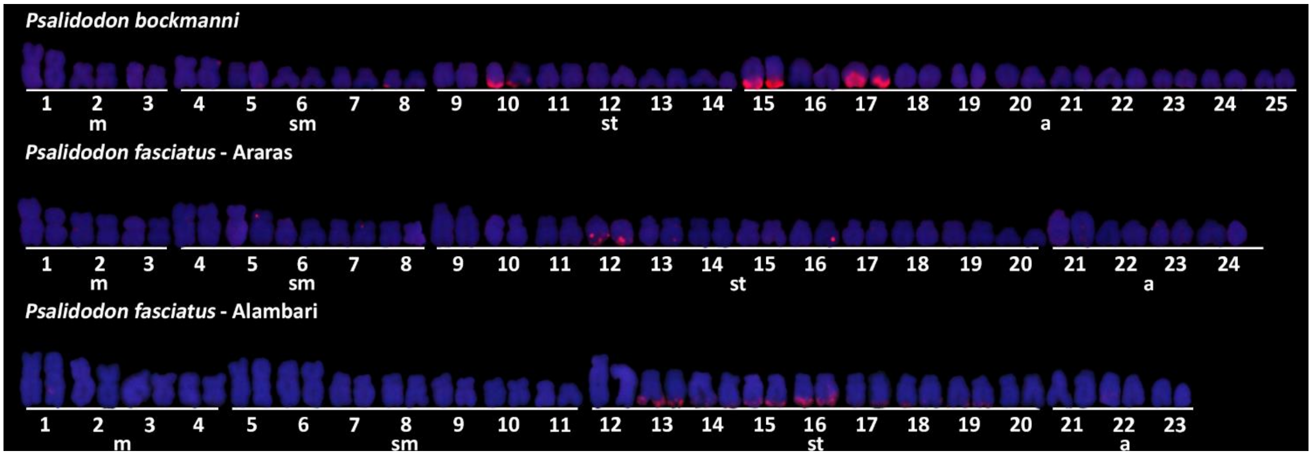

Supplementary Figure 10. Fluorescence *in situ* hybridization (FISH) mapping of ApaSat40-189 in *Psalidodon bockmanni*, *Psalidodon fasciatus* (Araras), and *Psalidodon fasciatus* (Alambari).

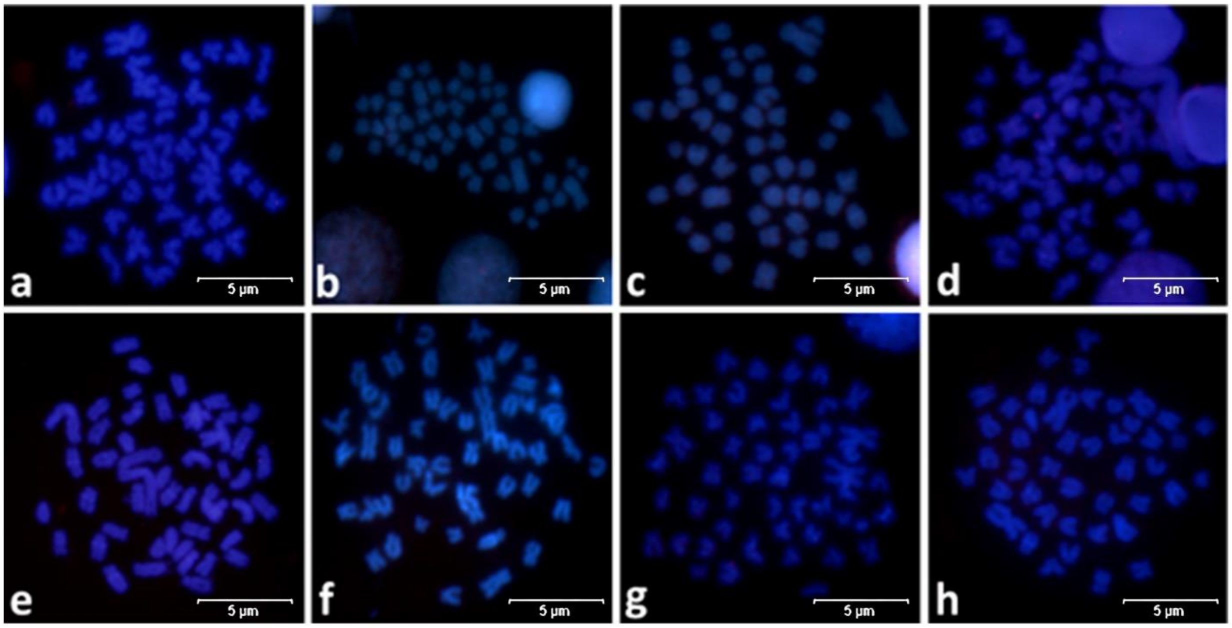

Supplementary Figure 11. Fluorescence *in situ* hybridization (FISH) mapping of ApaSat02-236 (a), ApaSat03-91 (b), ApaSat04-233 (c), ApaSat11-22 (d), ApaSat12-69 (e), ApaSat29-51 (f), ApaSat30-50 (g), and ApaSat40-189 (h) in *Astyanax lacustris*. Fish images were captured in a magnification of 1000×.

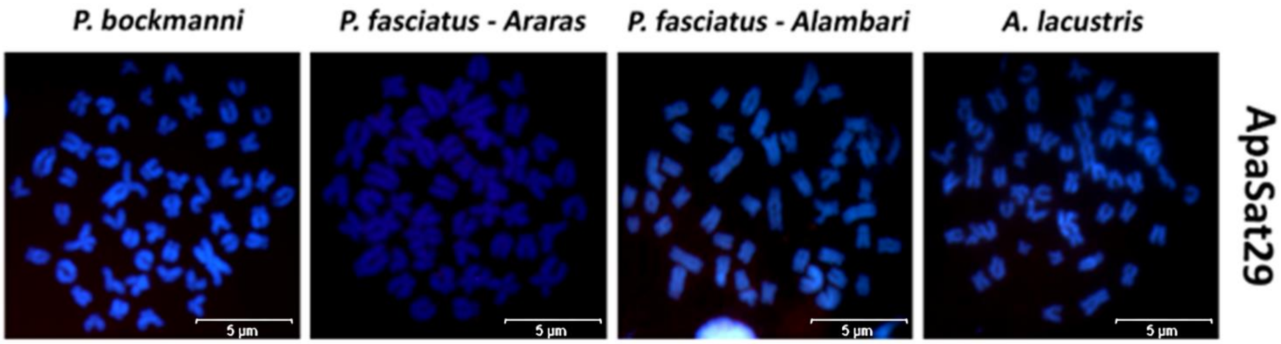

Supplementary Figure 12. Fluorescence *in situ* hybridization (FISH) mapping of ApaSat29-51 in *Psalidodon bockmanni*, *Psalidodon fasciatus* (Araras and Alambari), and *A. lacustris*. Fish images were captured in a magnification of 1000×.

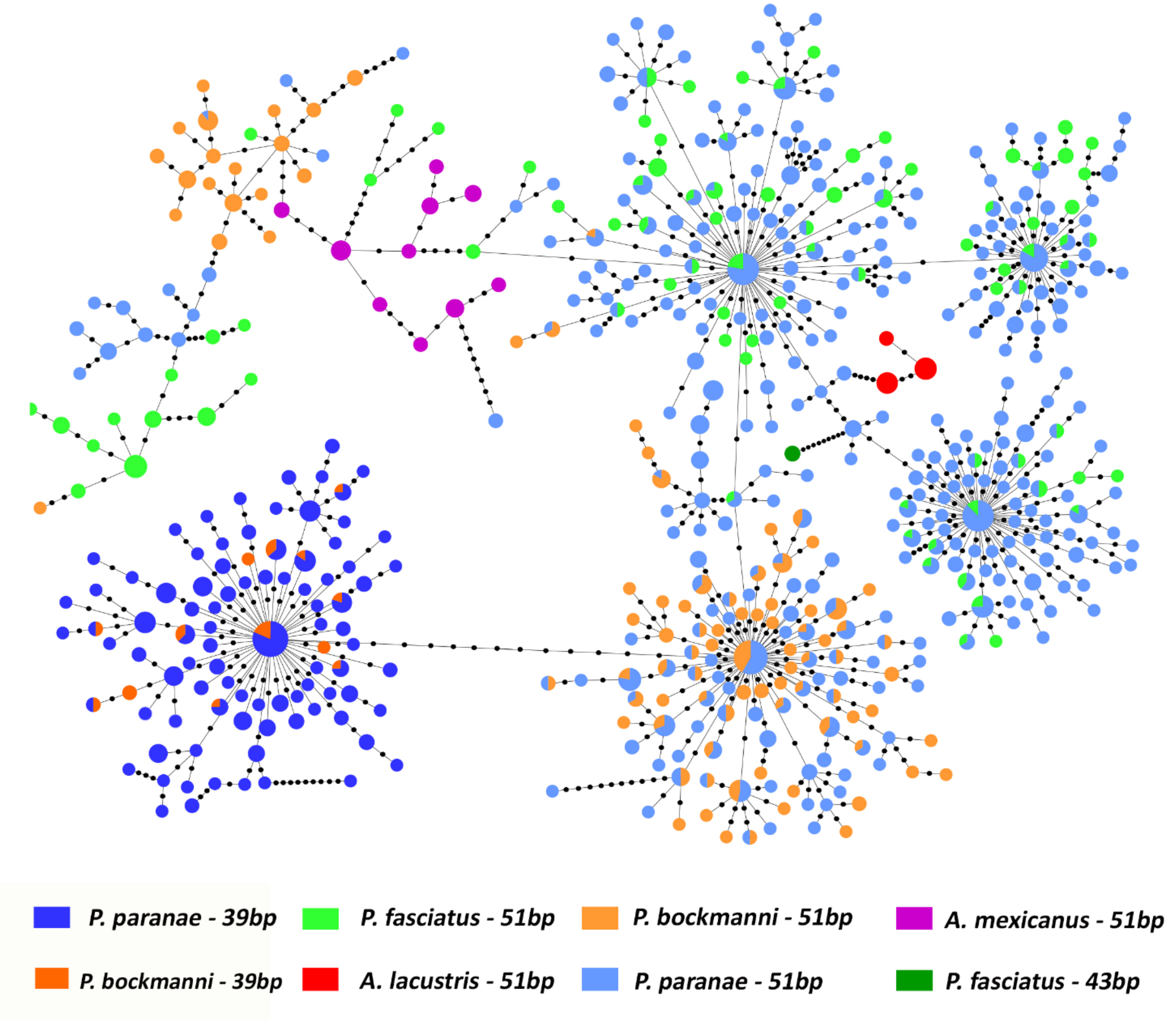

Supplementary Figure 13. Logarithmic minimum spanning tree (MST) demonstrating the haplotypes of variants of As51 in *Astyanax lacustris, Astyanax mexicanus, Psalidodon paranae, Psalidodon bockmanni,* and *Psalidodon fasciatus.* The term “bp” means base pairs.
